# Linking Yeast Gcn5p Catalytic Function and Gene Regulation Using a Quantitative, Graded Dominant Mutant Approach

**DOI:** 10.1371/journal.pone.0036193

**Published:** 2012-04-27

**Authors:** Amanda M. Lanza, John J. Blazeck, Nathan C. Crook, Hal S. Alper

**Affiliations:** 1 Department of Chemical Engineering, The University of Texas at Austin, Austin, Texas, United States of America; 2 Institute for Cellular and Molecular Biology, The University of Texas at Austin, Austin, Texas, United States of America; Michigan State University, United States of America

## Abstract

Establishing causative links between protein functional domains and global gene regulation is critical for advancements in genetics, biotechnology, disease treatment, and systems biology. This task is challenging for multifunctional proteins when relying on traditional approaches such as gene deletions since they remove all domains simultaneously. Here, we describe a novel approach to extract quantitative, causative links by modulating the expression of a dominant mutant allele to create a function-specific competitive inhibition. Using the yeast histone acetyltransferase Gcn5p as a case study, we demonstrate the utility of this approach and (1) find evidence that Gcn5p is more involved in cell-wide gene repression, instead of the accepted gene activation associated with HATs, (2) identify previously unknown gene targets and interactions for Gcn5p-based acetylation, (3) quantify the strength of some Gcn5p-DNA associations, (4) demonstrate that this approach can be used to correctly identify canonical chromatin modifications, (5) establish the role of acetyltransferase activity on synthetic lethal interactions, and (6) identify new functional classes of genes regulated by Gcn5p acetyltransferase activity—all six of these major conclusions were unattainable by using standard gene knockout studies alone. We recommend that a graded dominant mutant approach be utilized in conjunction with a traditional knockout to study multifunctional proteins and generate higher-resolution data that more accurately probes protein domain function and influence.

## Introduction

Establishing high-resolution, causative mapping of specific protein function and cell response is a critical facet underlying success in genetics, systems biology, drug discovery, and molecular biotechnology [1]. This task is challenging for multifunctional proteins that contain diverse functionalities including protein and DNA interactions and catalytic activity. These proteins play critical roles in epigenetic modification, signaling cascades, and transcriptional regulation. When relying upon commonly invoked approaches (such as gene deletions), it is difficult to directly link one particular function of these proteins (such as catalytic activity or a specific protein-protein interaction) to downstream gene regulation. The reason for this difficulty is that coarse modifications like knockouts remove the entire protein and thus all of its functions, thereby creating an environment for non-natural associations or activity compensations that confound data analysis. In this regard, gene knockout studies probe cellular response and compensation, not necessarily precise protein function. While alternative strategies to gene deletion have been used [2], [3], [4], [5], [6], [7], all of these methods result in pleiotropic effects that do not specifically isolate the multiple functionalities inherent in proteins. Here, we demonstrate the capacity of a unique, graded dominant mutant approach to enable the systems biology study of a yeast histone acetyltransferase.

The yeast histone acetyltransferase (HAT), Gcn5p, is a multifunctional protein with catalytic and binding domains (including Ada2 interaction and a bromodomain). A causative study of acetyltransferase activity thus requires a removal or reduction of catalytic function while maintaining native protein interactions. HAT proteins are important targets of genetic studies since they are critical for establishing acetylation of histones, which have long been recognized as a mark of euchromatin and an important activating genomic modification [8], [9]. The yeast gene, *GCN5*, encodes a histone acetyltransferase that serves as a well-studied prototype [10], [11], [12] for transcription-associated HAT activity. Gcn5p has a known crystal structure [13] and direct homologues in higher eukaryotic systems. Only a small number of Gcn5p putative gene targets have been identified even though it is presumed that this HAT globally controls gene expression [14]. Moreover, as this HAT is nonessential like many epigenetic factors, inherent protein redundancy implies that other HAT proteins may compensate for Gcn5p in its absence and thus confound data relying on knockout studies alone.

Some attempts have been made to specifically inhibit catalytic activity of similar epigenetic proteins through inhibitors including nucleotide analogues [15], [16] and other small molecules [17], [18], [19], [20]. However, inhibitors are difficult to design *de novo*, lack single target specificity, are limited in their concentration ranges, and often have a lower than anticipated response rate [17]. Classically, dominant mutations have been widely used to probe gene function, [21] improve tolerances and drug resistances, [22], [23], [24] characterize disease states, [25], [26], [27], [28] and map protein functional domains [29]. In this regard, small point mutations can abolish a particular function in isolation without disrupting other protein activities. Here, we exploit the inhibitory nature of dominant mutations and demonstrate that varying the expression level of a non-catalytic dominant mutant in the presence of the native, wild-type allele can specifically isolate and titer the catalytic activity of the wild type protein.

Despite the common use of dominant mutants, no prior study has paired these alleles with a promoter library to specifically and quantitatively grade an isolated protein function and collect systems level information. Here we demonstrate the power of a graded dominant mutant approach to isolate and causatively study the histone acetyltransferase catalytic activity of Gcn5p protein in the yeast *Saccharomyces cerevisiae* and in doing so, uncover previously unknown gene targets and functions of Gcn5p.

## Results

### gcn5-F221A competitively inhibits the catalytic function of Gcn5p in a dose-responsive manner

We first sought to study the influence of Gcn5p-based acetylation using a dominant mutant allele, *gcn5-F221A*, based on prior evidence of absent *in vitro* acetylation activity [30]. We observed that this allele failed to complement a BY4741 *gcn5* null strain *in vivo* (**Figure S1**) and determined it to possess no changes to Gibbs Free energy using Protein Interfaces, Surfaces and Assemblies [31], indicating a conservation of protein structure. Alongside these tests, a second catalytically inactive, dominant mutant (*gcn5-E173A*) was likewise constructed and tested (**Discussion S1**). This second mutant also competitively inhibited native Gcn5p acetyltransferase activity.

In order to create quantitative, graded expression of these dominant alleles, expression (and thus level of competitive inhibition) was modulated through the use of a promoter library. Expression of the mutant allele was established by cloning *gcn5-F221A* into centromeric yeast expression vectors under the control of a collection of mutant TEF-based promoters with previously established expression capacities [32], [33]. This library resulted in a ratio of mutant to wild-type expression ranging from 2.5 fold with the weakest promoter to 8–10 fold with the strongest promoter (**Fig. S2)**. Three genetic tests were used to establish and validate the gradation and competition of catalytic activity by this mutant.

**Figure 1:**
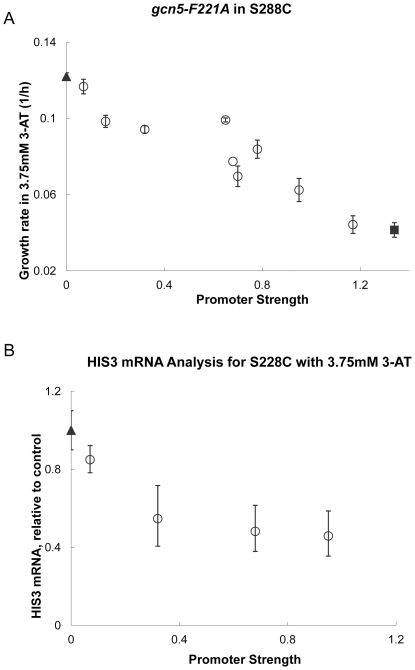
gcn5-F221A can impart a graded phenotype as detected by histidine starvation assays. The *gcn5-F221A* mutant was expressed in S288C with varying promoter strengths, and starvation response measured via growth rate in minimal media supplemented with 3.75 mM 3-aminotriazole. **a.** Growth rates of strains harboring *gcn5-F211A* (○) were compared to wild-type (▴) and *gcn5Δ* strains (▪). Error bars represent the standard deviation of biological triplicates. Increasing the expression level of *gcn5-F221A* (through progressively stronger promoters) results in a decrease in growth rate approaching the value of the knockout strain. **b.** *HIS3* mRNA levels were measured for select promoter strengths (.07, .32, .68 and .95) using RT-PCR. As *gcn5-F221A* promoter strength increases, *HIS3* expression decreases following a competitive inhibition pattern. These results demonstrate that *gcn5-F221A* can exhibit a graded, competitive phenotype at *HIS3* as measured by starvation response.

The first test involved the *HIS3* locus, a known acetylation target for Gcn5p [34]. Gene activation of *HIS3* by Gcn5p-based acetylation enables higher tolerance to a histidine analogue, 3-aminotriazole (3-AT). In a *gcn5Δ* strain, *HIS3* expression is decreased, leading to amino acid starvation in the presence of 3-AT and decreased cell growth. Each expression cassette controlling *gcn5-F221A* was transformed into *S. cerevisiae* S288C and growth rate was evaluated in the presence of 3-AT (**Fig. 1a**). Strains with low expression of the mutant allele most-closely resembled the wild-type strain, whereas at higher expression levels, strains resembled that of the *gcn5* null strain. Transcription of *HIS3* was found to decrease in a manner that followed a competitive inhibition curve (**Fig. 1b**). This data provides strong evidence that the *gcn5-F221A* allele competes for Gcn5p acetylation sites in the *HIS3* promoter region and effectively decreases *HIS3* transcription. While the growth rate trend (**Fig. 1a**) shows a clear correlation between mutant expression and growth rate, the trend is linear rather than an inhibition curve. We believe this arises from the more indirect measurement of growth rate, which is impacted by many cellular and environmental factors, and therefore integrates multiple signals, not just *HIS3* expression levels. By comparison, the measurement of *HIS3* mRNA levels (**Fig. 1b**) is a more direct measurement and thus presents the more expected competitive inhibition curve.

**Figure 2:**
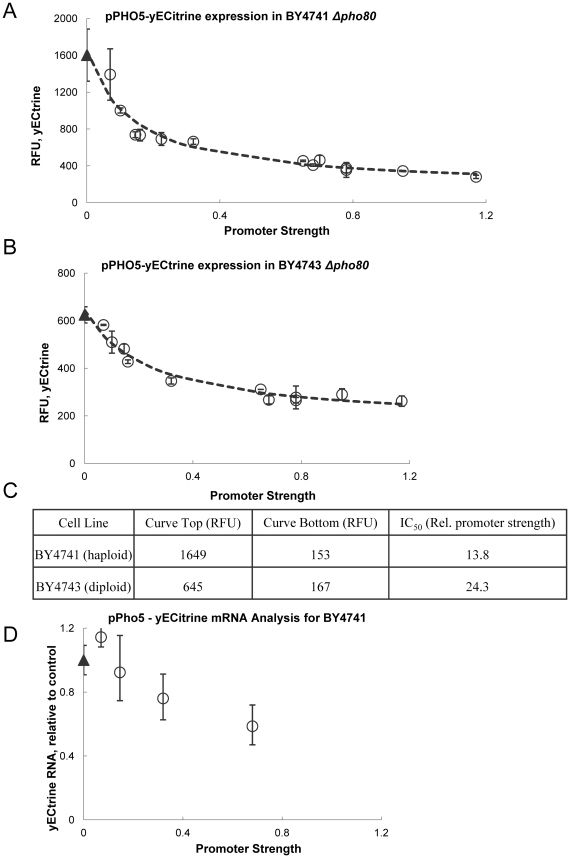
Evaluating competitive inhibition by gcn5-F221A using a synthetic pPHO5-yECitrine construct. The *gcn5-F221A* mutant was expressed with varying promoter strengths in **a.** the haploid BY4741 *pho80Δ* and **b.** the diploid BY4743 *pho80Δ*, and co-expressed with a second plasmid, containing the yECitrine gene driven by pPho5. Average fluorescence in mid-exponential phase are reported and error bars represent standard deviations of biological triplicates. Increasing expression of *gcn5-F221A* resulted in decreased mean fluorescence. The data was fit to a Hill-slope competitive inhibition model (dashed line) and IC_50_ values were extracted (**c**), indicating the relative promoter strength of *gcn5-F221A* resulting in half-maximal inhibition. Diploid yeast required nearly twice as strong promoter strength. **d.** yEcitrine mRNA levels were measured using RT-PCR for select promoter strengths (.07, .16, .32, and .70). The *gcn5-F221A* mutant serves as a competitive inhibitor to wild-type *GCN5* acetyltransferase activity.

A second test involved inhibiting the well-characterized Gcn5p-based regulation of the Pho5 promoter [35], [36], [37]. This test was conducted in two *pho80* knockout strains of yeast, a haploid (BY4741) and diploid (BY4743), as this mutation results in a constitutively active Pho5 promoter [36]. Both hosts contained an episomal synthetic gene circuit with the Pho5 promoter regulating expression of the fluorescent protein yECitrine. The activation of the Pho5 promoter was assayed in yeast strains harboring the collection of plasmids with graded *gcn5-F221A* expression (**Fig. 2a, b**). The fluorescent signal decreases with the expression level of *gcn5-F221A*, consistent with the hypothesis that this mutant allele directly competes with the native Gcn5p protein. We found that mean fluorescence followed a competitive inhibition model, where increased expression of the dominant mutant decreased the mean fluorescence.

We were able to fit our data to the Hill-slope competitive inhibition model:
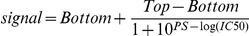
where *Top* is the signal strength in the absence of competition, *Bottom* is the signal strength of a competitively saturated system and *IC_50_*, or 50% effective concentration, occurs when the signal strength is reduced to the value halfway between the upper and lower bounds. The *signal* is a measure of average fluorescence in RFU and *PS* is the relative promoter strength of the dominant mutant. A best-fit was determined using a sum of least squares regression with the experimentally determined values. These models demonstrate that increased promoter strength is required to inhibit the two chromosomal copies of *GCN5* in a diploid strain (curve shown in **Fig. 2a and b**). Results of the IC_50_ values are found in **Fig. 2c**. Finally, yECitrine mRNA levels decreased as a function of *gcn5-F221A* expression (**Fig. 2d**). This test demonstrates the ability of a dominant mutant allele approach to make a direct measurement relating the grading of acetyltransferase activity to downstream gene expression (in this case, Gcn5p acetyltransferase activity and Pho5 promoter activity).

### gcn5-F221A competitively inhibits global histone acetylation at H3K18

**Figure 3:**
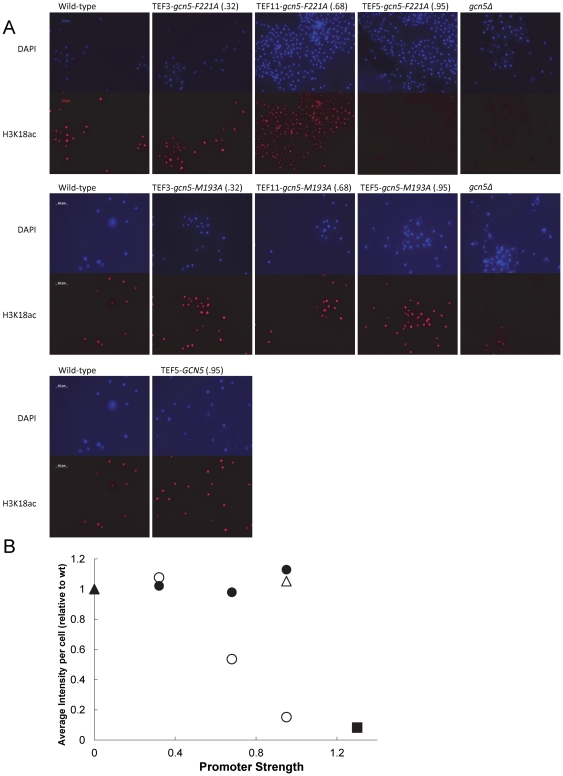
Global acetylation at H3K18 is attenuated by expression of mutant gcn5-F221A. Using immunofluorescence, H3K18 acetylation was assayed globally for strains harboring the *gcn5-F221A* mutant (○) expressed with varying promoter strengths (.32, .68, and .95). For comparison, *gcn5-M193A* mutant (•) (fully functional) with the same promoter strengths, along with wild-type (▴), *gcn5Δ* (▪) and wild-type with *GCN5* (Δ) over-expressed, were also examined. The primary antibody targets H3K18ac and the secondary antibody is an IgG tagged with DyLight 649. Cells were stained with DAPI to visualize nuclear material. **a.** Cells were imaged with both DAPI and Cy5 filters. The *gcn5-F221A* mutant results in global attenuation of H3K18ac and approaches *gcn5Δ* strain at high strength promoters. By comparison, the *gcn5-M193A* mutant and wild-type with *GCN5* result in no change to acetylation levels. **b.** Average cell intensity was quantified using Metamorph software and normalized relative to the wild-type.

In a third test, we sought to demonstrate that the graded dominant mutant, *gcn5-F221A*, was directly impacting histone acetylation. In *S. cerevisiae*, lysine 18 of histone 3 is primarily acetylated by Gcn5p, with very little acetylation occurring in a *gcn5Δ* strain [38]. An immunofluorescence assay for acetylated H3K18 residues was conducted using mid-exponential phase, fixed yeast cells. Three promoter strengths (0.32, 0.68, and 0.95 relative to wild-type TEF) were used to drive the expression of *gcn5-F221A* and these strains were compared to wild-type and *gcn5Δ* strains. Additionally, two further controls (over-expression of wild type *GCN5* and graded expression of a catalytically active mutant allele, *gcn5-M193A*) were used to demonstrate the specific acetylation inhibition only afforded by *gcn5-F221A*. Neither the wild-type *GCN5* nor the catalytically active mutant *gcn5-M193A* showed a change in global H3K18ac. By comparison, expression of the inactive, dominant mutants, *gcn5-F221A*, resulted in a dose-dependent decrease of H3K18 acetylation. These results, illustrated in **Fig. 3a** and quantified in **Fig. 3b**, demonstrate that *gcn5-F221A* competes directly with native Gcn5p, resulting in reduced histone acetylation. The second catalytically inactive, dominant mutant (*gcn5-E173A*) showed a similar direct impact on global H3K18ac (**Discussion S1**).

### Combining expression profiling with a graded dominant mutant approach reveals novel Gcn5p targets and function

**Figure 4:**
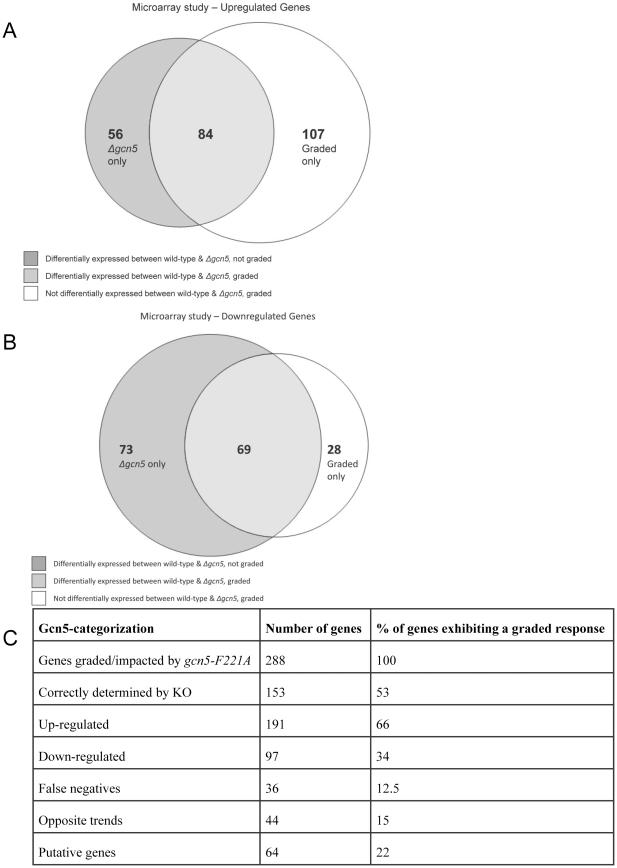
Expression analysis comparing a graded dominant mutant of gcn5-F221A to gcn5Δ. Microarray analysis was conducted for strains expressing the *gcn5-F221A* mutant at varying promoter strengths (.32, .68, and .95) along with wild-type and *gcn5Δ* cells. **a.** Of the genes found to be up-regulated compared to the wild-type, only 84 were commonly identified by both the dominant mutant and knockout, and 107 were only identified by the dominant mutant. **b.** Significantly fewer genes were found to be down-regulated, of which only 69 were commonly identified and 28 were only identified by the dominant mutant. **c.** Characterization of the 288 genes observed to exhibit a graded response with respect to increasing levels of *gcn5-F221A*.

Next, we sought to evaluate the global influence of Gcn5p acetyltransferase activity on yeast gene expression. By using our approach, genes whose expression changes as a function of *gcn5-F221A* level are changing as a result of decreased acetyltransferase activity. Using microarrays, we identified and classified differentially expressed genes between *S. cerevisiae* (S288C) wild-type, the *gcn5* null strain, and mutant *gcn5-F221A* expressed at three different promoter strengths (0.32, 0.68 and 0.95) (**Table S1**). A total of 282 genes were found to be differentially expressed (p-value<0.05, abs(log_2_)>1) between the wild-type and knockout strain. This dataset overlaps a similar previously reported gene expression study for *gcn5Δ* with 98% coverage [39]. A total of 288 genes were found to be differentially graded in response to *gcn5-F221A* (i.e. genes whose expression changes monotonically in response to *gcn5-F221A* and all of which had p-values<0.05). Despite these similar numbers, only 153 genes (53%) found in the knockout data set overlap with the graded dominant mutant dataset (**Fig. 4c**). This initial analysis indicates that, for multifunctional proteins, classifying genes and regulation based exclusively on knockout data is misleading. Of the 288 genes influenced by *gcn5-F221A*, 66% increased in expression in response to increasing *gcn5-F221A* levels (**Fig. 4a**) whereas the *gcn5* null strain significantly overestimates the number of under-expressed genes (**Fig. 4b**). This data set augments our knowledge of gene targets regulated by Gcn5p-acetyltransferase activity.

**Figure 5:**
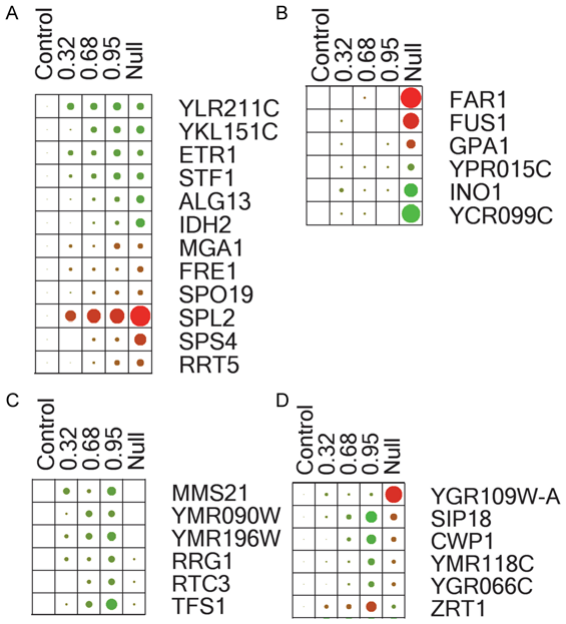
Gene expression heat maps for select genes illustrating unique traits in this study. Four key (non-mutually exclusive) trends in gene expression were observed in this study: catalytically associated genes, false negatives (compared to a knockout), non-catalytically associated genes, and opposites. In the heat map, red indicates underexpression, and green overexpression relative to the control, and the size of the dot is proportional to magnitude of expression. A select sample of genes was used to display the four trends. **a.** Catalytically associated genes have expression that changes (either up or down compared to control) as a function of *gcn5-F221A*. The threshold for response varies for each of these genes. **b.** 129 non-catalytically associated genes (changed in *gcn5Δ*, but not graded by *gcn5-F221A*) were identified, suggesting these targets are not impacted by Gcn5p acetylation, but perhaps by another indirect effect of Gcn5p such as protein complex association. **c.** 36 false negative genes were identified, in which expression is graded by the dominant mutant, but are unchanged in the knockout strain. These genomic loci are most likely directly acetylated preferentially by Gcn5p along with other compensatory HATs. **d.** An additional set of 44 genes demonstrate an opposite effect in the presence of the dominant mutant compared to the *gcn5Δ*.

Four non-mutually exclusive classifications of gene expression were used to characterize the targets found in this study—catalytically associated, non-catalytically associated, false negatives (compared to a knockout), and opposites—by comparing these gene targets to data obtained using the traditional knockout approach. A subset of Gcn5p-impacted genes illustrates these trends (**Fig. 5**). The 288 genes identified through *gcn5-F221A* inhibition of native Gcn5p acetylation display a ‘graded’ response and are therefore associated with Gcn5p catalytic activity. Within this classification, variations in the response to level of gradation exist. Some genes (such as *ETR1* and *YLR211C*, **Fig. 5a**) achieve maximal gradation (a plateaued response matching that of a knockout condition) at low levels of *gcn5-F221A*. We posit that similarly responding genes (with low grading thresholds) are strongly impacted by Gcn5p acetylation and potentially have the fewest redundant epigenetic modification mechanisms in yeast. In contrast, genes that require higher levels of the dominant mutant to achieve maximal gradation (such as *IDH2* and *RRT5*, **Fig. 5a**) are less sensitive to acetylation by Gcn5p or have more redundant regulation mechanisms. This approach allows for an evaluation of gene thresholding responses, an important concept in systems biology modeling.

We observed 129 ‘non-catalytically associated’ genes in this data set (genes differentially expressed in the knockout strain, but not significantly impacted in response to the graded dominant mutant) (**Fig. 5b**). Since *gcn5-F221A* only inhibits acetyltransferease activity, we hypothesize that these non-catalytically associated genes are not influenced by Gcn5p acetylation activity, but instead are influenced by another indirect effect of Gcn5p, such as protein complex association. Furthermore, we observed that 36 (12.5%) of those genes impacted by the dominant mutant showed no change in expression between the wild-type and knockout strains (**Fig. 4c** **,** **5c**). These ‘false negatives’ are clearly impacted by Gcn5p activity, and we hypothesize that these genomic loci are directly acetylated by Gcn5p, but in its absence, another HAT with redundant functionality steps in.

Finally, we observed that 44 genes (15%) impacted by the dominant mutant display an ‘opposite’ effect in expression as predicted by the gene knockout (**Fig. 4c** **,** **5d**). In the majority of these cases, these genes were over-expressed in response to *gcn5-F221A* but significantly decreased in expression in the *gcn5Δ* strain.

The impact of these various gene classes was further evaluated using RT-PCR for select genes exhibiting a graded response. We used additional promoter strengths to allow for a more quantitative, high-resolution measurement of the impact of Gcn5p catalytic activity (**Discussion S2**). Additional control samples were used to demonstrate that these genes had altered expression as a result of *gcn5-F221A* only, and not in the presence of over-expressed *GCN5*, which at the genetic level behaved the same as wild-type yeast (**Fig. S3**). Moreover, these higher-resolution datasets linking catalytic activity and gene regulation enable a more precise measurement of thresholding effects.

Finally, we sought to determine what, if any, impact varying expression of mutant Gcn5p had on the expression of native Gcn5p. A p415-pGcn5-yECitrine plasmid was constructed, with both a short and long *GCN5* promoter, and co-transformed with the p416-TEF_x_-*gcn5-F221A* plasmid collection. In this system, fluorescent protein expression is controlled by the *GCN5* promoter, thus this construct serves as a promoter-based transcription reporter. Using mid-exponential, biological triplicates and flow cytometry, we measured fluorescent levels across the full range of mutant *gcn5-F221A* expression. Regardless of promoter strength driving mutant *gcn5-F221A*, we observed no change in fluorescent expression (**Fig. S4**). This result indicates that expression of *gcn5-F221A* does not create artificial feedback or perturbations of native *GCN5* expression. Thus, these results demonstrate the clear link between the data we observe and the lack of catalytic function inherent in *gcn5-F221A*.

### Histone modifications are accurately uncovered using a graded dominant mutant approach

We next sought to see whether specific chromatin modifications can be deduced from microarray data alone when using a graded dominant mutant approach. To do so, genes identified in our microarray study were analyzed using Chromatin DB [40] (**Discussion S3**). Using genes identified by a *gcn5Δ* knockout (including subclasses of up-regulated, down-regulated, and differentially expressed), no significant enrichment or depletion of chromatin lysine acetylation is evident. This same lack of enrichment or depletion is observed using the microarray data obtained by a separate and independent *gcn5Δ* study [39]. However, by examining the graded up genes with low grading thresholds identified in this study, significant depletion is seen in H2BK11ac, H2BK16ac, H3K18ac, H3K14ac, and H3K23ac with p-values of less than 10^−3^ to 10^−4^. This profile of histone modifications mimics those observed in a Gcn5p binding study [11]. Furthermore, the ‘false negative’ gene set exhibits acetylation depletions for the same lysine residues as genes that are graded up. This clearly demonstrates that ‘false negative’ genes are indeed direct targets of Gcn5p acetylation and explains why the vast majority of these ‘false negative’ genes increase in expression in response to *gcn5-F221A*. In contrast, the non-catalytically associated data set exhibits no enrichment or depletion of chromatin lysine modifications. In the case of those genes exhibiting an ‘opposite’ response, the primary histone modification that is observed is a depletion of H4K16ac (p-value <10^−3^). It is well known that Sir2p and Esa1p are responsible for targeting H4K16 [41], which implicate the actions of these proteins as potential compensators for Gcn5p. Collectively, these results demonstrate that the graded dominant mutant approach can identify the canonical acetylation targets of Gcn5p [42].

### Gene ontology analysis reveals new cellular processes that are impacted by Gcn5p acetylation

Gene ontology and network analysis tools were used to further classify the genes influenced by *gcn5-F221A* activity and evaluate the dataset (**Discussion S4**). Three functional classes (nucleolus, ribosome biogenesis, and RNA metabolic processes) were significantly enriched in the set of genes exhibiting an under-expression graded response. Nearly 70% of the genes exhibiting under-expression were associated with these functional classes. Furthermore, one gene ontology class (oxidoreductase activity) was overrepresented in genes exhibiting an over-expression in a graded fashion, and is thus a target for Gcn5p-based gene repression. This analysis expands the role of Gcn5p activity to other fundamental cellular processes.

### Graded dominant mutant approach can interface with other phenotypic and genetic assays

Finally, we sought to demonstrate how the graded dominant mutant approach can be used in conjunction with phenotypic and genetic assays. Prior to this work, it was unclear whether acetylation or protein-protein interaction is the root cause of *gcn5Δ* synthetic lethal genes. To address this issue, we paired a gene deletion with various promoter strengths driving *gcn5-F221A* to simulate the lethal double knockout strain in the haploid yeast BY4741. Twenty two of these synthetic lethal genes were selected for this study and evaluated (**Table I** **, Table S2**). Only three gene knockouts, *Δccr4*, *Δrsc2* and *Δrtt109*, were highly impacted in a graded fashion by the dominant mutant. *Δrtt109*, a HAT known to acetylate H3K56 and H3K9 [43], demonstrated the most significant impact. Fifteen of the gene deletions were moderately impacted and four showed almost no change. Collectively, these results implicate the relative importance of Gcn5p's catalytic activity versus its protein and DNA interactions. As a comparison, ten BY4741 null strains were selected at random to serve as a control group (**Table S2**). None of these strains showed a growth-rate dependent response to the *gcn5-F221A* mutant, indicating the significance of the results described above.

**Table I:** Impact of gcn5-F221A on the growth rate of GCN5 synthetic lethal genes.

*GCN5* synthetic lethal	Influence of acetyltransferase activity
*ccr4* *rtt109* *rsc2*	Highly Impacted
*eaf7* *hhf2* *hht2* *hsl1* *hsl7* *leu2* *mot2* *nam2* *not5* *paa1* *pap2* *rad6* *rpd3* *sin3* *snf2*	Moderately Impacted
*elp3* *iki3* *pho23* *spt20*	Not Impacted

We examined 22 gene knockouts with known synthetic lethal interactions to *gcn5* null. The *gcn5-F221A* dominant mutant was expressed at varying levels in the background of a knockout strain and growth rate was measured (**Table S2**). Three gene knockouts, *Δccr4*, *Δrsc2*, and *Δrtt109*, are highly impacted by the *gcn5-F221A* mutant and exhibited a more than a 20% reduction in growth rate when the *gcn5-F221A* mutant was highly expressed, indicating these synthetic lethal pairings are highly dependent on Gcn5p catalytic activity. The majority of the genes show a moderate decrease in growth rate as mutant expression is increased while several showed no growth rate changes. Most of these synthetic lethal interactions are not impacted by catalytic activity, but rather require Gcn5p for a protein-protein or protein-DNA interaction. Ten gene deletions were randomly selected as a control and none exhibit any response (**Table S2**).

Finally, we sought to investigate the global impact of Gcn5p acetyltransferase activity on cellular phenotypes. To do so, we evaluated the impact that grading this activity has on the basis of documented large-scale chemical tolerance assays of null mutants. Yeast strains with a *gcn5* null allele have previously been shown to have increased sensitivity to cycloheximide [44], ethanol [44], 5-fluorouracil [45], KCl [46], MnCl_2_ [46], CaCl_2_ [46], and sulfanilamide [47]. Growth inhibition assays were performed using strains containing gradations of the dominant mutant, as well as a wild-type control and a *Δgcn5* control, as described in the **Materials and Methods**.

When treating the strains with cycloheximide, we observed a graded, linear decrease in growth rate that coincided with increasing *gcn5-F221A* expression, showing that cycloheximide tolerance is controlled by Gcn5p acetyltransferase activity (**Fig. S5**). Assays performed using ethanol, 5-fluorouracil, KCl, 4mM MnCl_2_, and 8 mM MnCl_2_ as a growth inhibitor did not exhibit this trend (**Table S3**). These growth inhibitors are akin to the non-catalytically associated gene expression data set, and we hypothesize that increased sensitivity to these growth inhibitors is not a result of decreasing cellular Gcn5p acetylation, but by a separate, indirect effect. Despite prior reports, sulfanilamide, CaCl_2_, and 40 mM MnCl_2_ inhibitors did not impact growth rate for any of the strains in our liquid-culture based experiment.

## Discussion

Using a graded dominant mutant approach and Gcn5p as a case study, we are able to determine global gene targets and impacts, and to extract the causative linkage between the catalytic domain of Gcn5p and gene regulation. In particular, we (1) find evidence that Gcn5p is more involved in cell-wide gene repression, instead of the accepted gene activation associated with HATs, (2) identify previously unknown gene targets and interactions for Gcn5p-based acetylation, (3) quantify the strength of some Gcn5p-DNA associations, (4) demonstrate that this approach can be used to correctly identify canonical chromatin modifications, (5) establish the role of acetyltransferase activity on synthetic lethal interactions, and (6) identify new functional classes of genes regulated by Gcn5p acetyltransferase activity—all six of these major conclusions were unattainable by using standard gene knockout studies alone. These results demonstrate the power of the graded dominant mutant approach, which unlike traditional methods, only impacts one particular facet of the querying protein (in this case, acetyltransferase activity) and is therefore especially useful for studying multifunctional proteins and global regulators.

Despite the common conception that Gcn5p-based acetylation is gene activating, we posit that Gcn5p-based acetylation serves a dominant role in maintaining global gene repression in yeast. We found that over-expression of a catalytically inactive dominant mutant led to up regulation of 66% of affected genes. This finding is unexpected and not evident from traditional knockout experiments, as gene expression changes in the knockout strain were equally distributed between over and under expression. This is the first time that Gcn5p-based acetylation has been implicated with global gene repression, and may be a direct function of Gcn5p or an indirect result of additional gene regulators that are controlled by Gcn5p.

Our global microarray study identifies a set of 44 ‘opposite’ genes, whose expression in the presence of the catalytically inactive dominant mutant is opposite that of expression in a *gcn5Δ*. Further analysis of this set of genes indicates that nearly half are shown to be associated with the SAGA complex in an independent study [39]. It is likely that this ‘opposite’ phenomena is due to the partitioning of Gcn5p function and targeting across the domains (potentially the catalytic and bromodomains). In the case of the graded dominant, targeting of the SAGA complex can still occur and thus transcription is increased at these genes. However, in a gene knockout, the entire Gcn5p transcriptional coactivator is missing and thus transcription is impeded significantly. Underacetylated H4 histone proteins have also been shown to have a biased association with SAGA-regulated genes [39], further solidifying the SAGA-complex link to these opposite genes. These results provides another example that removing a globally functioning protein like Gcn5p results in an artificial genetic background with misleading observations regarding true protein-DNA interactions. Moreover, these results highlight how novel hypotheses of function can be deduced from this approach.

Based on the results presented here, we would recommend that a graded dominant mutant approach be utilized in conjunction with a traditional gene knockout to study gene regulatory proteins, especially those that serve multiple functions. The resulting data is higher-resolution and more accurately defines protein domain function and influence. While demonstrated here for the case of acetyltransferase activity of the yeast protein Gcn5p, this approach can theoretically be extended to other proteins and domains of interest. By creating two distinct Gcn5p dominant mutants that could both be graded and competitively inhibit native Gcn5p, we demonstrated that this approach is easily implemented. This approach uniquely enables a systems biology view of the cell while at the same time leveraging synthetic biology tools [48]. The identification of dominant mutations that can remove single functions are either well-documented for many proteins of interest or can be identified with the proper genetic screens. Additionally, promoter libraries with documented expression capacity are available for most major model systems [32], [49], [50]. Thus, this approach is generalizable for other proteins in classes such as epigenetic modification, signaling cascades, and transcriptional regulation as well as for essential genes, which cannot be deleted, and this method is not necessarily restricted to the yeast system studied here. In addition, this approach can be combined with any cell state assay including, but not limited to, gene expression analysis, phenotypic assays, genetic screens, ChIP analysis, and metabolomics. In conclusion, the graded dominant mutant approach is able to circumvent the problems seen in standard genetic approaches and can provide a causative linkage between specific protein function and phenotype.

## Materials and Methods

### Strains and Plasmids

Yeast expression vectors were propagated in *Escherichia coli* DH10β (**Table S4**). All experiments were carried out in *Saccharomyces cerevisiae*, with parent strains including BY4741, BY4743, and S288C and their derivatives described in **Tables S4** and **S5**. The BY4741 knockout strains were provided by the Marcotte laboratory (University of Texas at Austin, ICMB). S288C and BY4743 homozygous *Δpho80/Δpho80* strains were purchased from OpenBiosystems. The S288C Δ*gcn5* strain was made by replacing the wild-type *GCN5* gene with a hygromycin-B resistance gene amplified from plasmid pAG32 using primers 1 and 2 (**Table S6**) and extended using primers 3 and 4, for a final fragment with 80 base pairs of genomic homology both upstream and downstream. Using a high efficiency yeast transformation protocol [51], 1 µg of fragment was transformed into competent S288C cells and were plated on YPD supplemented with 100 µg/mL hygromcyin-B. The genotype of the S288C Δ*gcn5* strain was confirmed by extracting genomic DNA and performing both a positive PCR control (primers 5–8) and a negative PCR control (primers 9 and 10).

The wild-type *GCN5* gene was amplified from BY4741 gDNA using primers 9 and 10 and cloned into the pUC19 vector using restrictions enzymes XbaI and SalI. After confirming the accuracy of the *GCN5* sequence, mutations M193A, F221A and E173A were introduced using the Stratagene Quikchange mutagenesis kit and primers 11 to 14, and 39 and 40. The mutant GCN5 genes, as well as the wild-type gene, were cloned into the library of p416-TEF_mutant_ vectors [32] using the XbaI and SalI restriction enzymes. The *gcn5-M193A*, *F221A*, and *E173A* plasmid collections were transformed into BY4741 *Δgcn5* using a Gietz lithium acetate protocol [51] and selecting on drop out media deficient in uracil to create strains AML1 through AML30 (**Table S5**).

To allow for expression in amino acid free media, the p416-TEF_mutant_-*gcn5-F221A* plasmid collection was modified to include a G418 resistance gene. Using primers 15 and 16, the gene was amplified from the pUG6 plasmid. The p416-TEF_mutant_-*gcn5-F221A* plasmid collection and the resistance gene were digested with StuI and EcoRV. The new p416-TEF_mutant_-*gcn5-F221A*-G418 plasmid collection was transformed into S288C using a Gietz lithium acetate protocol and selected on YPD plates supplemented with 200 µg/mL G418 to create strains AML31 through AML40 and AML227–228. This process was repeated to create p416-TEF_mutant_-*gcn5-E173A*-G418 and p416-TEF_mutant_-*gcn5-M193A*-G418 plasmid collections, and to create strains AML229–247.

The p415-pPho5-yECitrine plasmid was constructed for fluorescence assays. The *PHO5* promoter, shown to be contained in the thousand base pairs upstream of *PHO* [35], was amplified using primers 17 and 18. Using restriction enzymes SacI and XbaI, pPho5 was cloned into the p416-TEF-yECitrine vector, replacing the TEF promoter in front of the yECitrine fluorescence gene. Using SacI and KpnI, the pPho5-yECitrine fragment was moved to the p415 plasmid, which contains a leucine auxotrophic marker. Along with the p416-TEF_mutant_-*GCN5* plasmid collections, the p415-pPho5-yECitrine plasmid was transformed into BY4741 Δ*pho80* and BY4743 Δ*pho80* using a Gietz lithium acetate protocol and selected on drop out media deficient in both uracil and leucine to create strains AML51 through AML66. The p415-pGcn5-yECitrine plasmid was constructed for fluorescence assays. The *GCN5* short promoter (350 bp) was amplified using primers 41 and 42, and the long (640 bp) promoter with primers 42 and 43 directly upstream from the *GCN5* gene. Using SacI and XbaI, the p415-pPho5-yECitrine plasmid was replaced with p415-pGcn5-yECitrine, and then along with the p416-TEF_mutant_-*GCN5* plasmid collection, transformed into BY4741 Δ*pho80* using a Gietz lithium acetate protocol and selected on drop out media deficient in both uracil and leucine to create strains AML248 through AML261 (**Table S5**).

Twenty-two BY4741 single gene knockouts, corresponding to genes that form a synthetic lethal phenotype with *gcn5Δ*, were transformed with a p416-TEF control plasmid, and p416-TEF_mutant_-*gcn5-F221A* plasmids with promoter strengths of 0.16, 0.32, 0.68 and 0.95. A Gietz lithium acetate protocol was used and colonies were selected in triplicate from drop out media deficient in uracil to create strains AML67 through AML176. An additional ten BY4741 single gene knockouts, selected at random, were transformed under identical conditions to serve as an experimental control (AML177–226).

All strains listed above were selected and tested in biological triplicate at minimum. Some strains and assays were tested with up to 6 biological replicates.

### Media and Growth Conditions

YPD media contains 20 g/L yeast extract, 10 g/L peptone and 10 g/L glucose. Minimal media for S288C strains contains 6.7 g/L nitrogen base, 20 g/L glucose and 200 ug/mL G418. Minimal media for BY4741 and BY4743 strains was supplemented with amino acids; 0.77 g/L of CSM –Ura (MP Biomedicals) for p416 vectors and 0.67 g/L of CSM –Leu –Ura (MP Biomedicals) for p416/p415 vectors. Media M-gYG418 contained 20 g/L glucose, 6.7 g/L YNB, and 200 µg/mL antibiotic G418. Bacteria were grown in lysogeny broth with ampicillin. All yeast strains were grown at 30°C and bacteria at 37°C. Agar plates were grown in standing incubators and cultures in shakers operating at 225 rpm. Passage numbers for yeast cultures were kept low (2–3) for all experiments.

### Growth Experiments

Complementation studies were conducted using strains AML1- AML30 and AML228–241. From stationary phase culture, a honey-comb plate was inoculated in triplicate with a starting OD of 0.1. Minimal media lacking uracil was supplemented with 3-aminotriazole. Using a Bioscreen C Growth Curve Analysis System, optical density measurements were taken every ten minutes for 24 hours. Temperature was maintained at 30°C and continuous, high shaking was used. Growth rate was calculated as the slope of the natural log of optical density versus time during the exponential growth phase. The histidine starvation assay was conducted using strains AML31 through AML40. From stationary phase culture, a honey-comb plate was inoculated with 4–6 biological replicates with a starting OD of 0.1. A minimal media composed of glucose, yeast nitrogen base without amino acids, 3.75 mM 3-aminotriazole and 200 ug/mL G418 was used. S288C wild-type and S288C Δ*gcn5*, both transformed with an empty p416-TEF-G418 plasmid, served as controls. Optical density measurements were collected over a period of 30 hours using a Bioscreen C and settings described previously.

Ten additional growth inhibition assays were conducted, in which a total of 50 strains were assayed, including the above controls, and 4–5 biological replicates of strains AML31 through AML40. The 50 strains were grown to stationary phase in 3 mL of M-gYG418 and then a honey-comb plate was inoculated with a starting OD_600_ of 0.1 in 250 µl fresh M-gYG418 either with or without (control cultures) a putative Gcn5p-dependent growth inhibition additive (**Table S3**). Optical density measurements were collected for the 100 cultures over a period of 60 hours using the Bioscreen C.

### Fluorescence Assays

Fluorescence assays were conducted using strains AML41 through AML66. 4–6 biological replicates were grown in drop out media lacking uracil and leucine until stationary phase. Fresh cultures were seeded at a low starting optical density (approximately 0.005) and allowed to grow to early exponential phase. The cell mass was collected by centrifugation and re-suspended in ice cold water. Fluorescent expression profiles were determined using a FACS Calibur and compared to a control population. Forward scattering had a voltage setting of E00 and ampgain of 2.96, side scattering a voltage of 505 and ampgain of 1.00 and fluorescence a voltage of 551 and ampgain of 1.00. Forward and side scattering data were linear and fluorescence was collected on a logarithmic scale. Threshold was set to a forward scattering value of 52. An average fluorescence and standard deviation was calculated from the mean values for the biological replicates. The competitive binding curve was determined for both the haploid and diploid by identifying parameters such as to minimize the sum of squares error for each data set.

Fluorescence assays were conducted using strains AML248 through AML261. Three biological replicates were grown in drop out media lacking uracil and leucine and allowed to grow to early exponential phase, and prepared as previously described above. Fluorescent expression profiles were determined using a FACS Fortessa and compared to a control population. Forward scattering had a voltage setting of 209 and ampgain of 1.00, side scattering a voltage of 209 and ampgain of 1.00 and fluorescence a voltage of 308 and ampgain of 1.00. Forward and side scattering data were linear and fluorescence was collected on a logarithmic scale. Threshold was set to a forward scattering value of 5000 with an OrOperator and area scaling of 0.71.

### Yeast Immunofluorescence

Global histone acetylation at H3K18 was measured using yeast immunofluorescence. Strains AML32, 34, 39, 227, 228, 242–247, 262 were grown to mid-exponential phase and fixed by adding a 10^th^ volume of 37% formaldehyde for 2 hours. Cells were washed twice with PBS and resuspended in 500 µl of a spheroplasting buffer (1.2 M sorbitol and 0.1 M KH_2_PO_4_ at pH of 7.5). Cells were stored for 1–2 days at 4°C. Spheroplasts were made by incubating 200 µl of fixed cells with 1.2 µl of zymolase (Zymo Research) and 3.2 µl of β-mercaptoethanol for 30 minutes at 30°C. Spheroplasts were washed once with 1 mL of PBS+0.05% Tween 20 and resuspended in 100 µl of PBS+0.05% Tween 20. Slides were treated with 50 µl of 1 mg/mL poly-L-lysine (>400,000 MW) for 15 minutes, followed by 3 water washes. After the slides were completely dry, 20 µL of spheroplasts were added to each well for 5 minutes, followed by 3 PBS washes. The slide was immersed in ice cold methanol for 5 minutes and ice cold acetone for 30 seconds. After drying, the slide was rehydrated by adding 50 µl of PBS for 5 minutes, followed by a PBS wash. A blocking solution composed of PBS and 1 mg/mL BSA was added (20 µL) to each slide followed by 30 minutes in a humid chamber. The slide was then washed 3 times with PBS. 20 µl of H3K18ac primary Rabbit antibody (Abcam) diluted 500-fold in blocking solution was added to each slide and incubated for 90 minutes. The slide was washed 3 times with PBS. 20 µl of anti-Rabbit Goat IgG DyLight 649 secondary antibody (Abcam) diluted 200-fold in blocking solution was added to each slide and incubated for 90 minutes in the dark. The slide was washed 3 times with PBS. 20 µl of 1 µg/mL DAPI in PBS was added to each well for 5 minutes, followed by 3 washes with PBS. A drop of fluorescent mounting medium (KPL) was added to each slide along with cover glass (#1.5 thickness) before sealing with nail polish. Slides were imaged using the Zeiss Axiovert instrument and a 100× magnifying lens. The DAPI and Cy5 filters were used respectively to image DAPI and DyLight 649 staining. Average intensity per cell was determined using Metamorph software.

### TEF Promoter Engineering

Additional variants of a weak TEF promoter (TEFpmut7) [32] were generated via error-prone PCR using the Genemorph II Random Mutagenesis Kit from Stratagene and primers 33 and 34. Six reactions containing differing template concentrations were combined to create 2 libraries of differing error rates (**Table S7**). Libraries were cleaned using the QIAquick PCR Purification Kit and cut with SacI and XbaI restriction enzymes (New England Biolabs). Fragments were ligated into a yeast expression vector upstream of the yECitrine fluorescent gene. 150 ng of each ligation was transformed into competent *E. coli* and plated onto LB agar plates containing 100 µg/mL ampicillin. For each library, approximately 17,500 colonies were scraped and collected in liquid culture and diluted to an optical density of 6 using LB media, and plasmid DNA was extracted using a Qiagen miniprep kit. From each plasmid library, 50 ng DNA was transformed [52] into *S. cerevisiae* BY4741 and plated on drop out media deficient in uracil.

110 yeast colonies were isolated from the libraries and grown in minimal media deficient in uracil to an optical density of 0.5 and analyzed by FACS Calibur, compared to a control strain. Mutants displaying fluorescence between 30% and 2% of the control population and low cell-to-cell variability were isolated, and plasmid DNA was extracted using the Zymoprep Yeast Plasmid Miniprep I. These plasmids were then sequenced (primers 35 and 36, **Fig. S6**), and retransformed into yeast to confirm promoter strength. The selected promoters (Tef32, 51 and 77) have strengths of 0.10±0.01, 0.15±0.01, and 0.22±0.02 relative to a native TEF promoter.

### Real Time PCR

Relative transcription levels were quantified using real time PCR from whole cell RNA extracts. Cell lines were grown in minimal media (M-gYG418) with a starting optical density between 0.004 and 0.005 until they reached a density between 0.4 and 0.5, at which point whole cell RNA was extracted using Ambion's Ribo-Pure kit for yeast. RNA quantification was performed with a Nanodrop 2000.

The S288C cell lines used for the analysis of *HIS3* mRNA levels were grown in media supplemented with 3.75 mM 3-aminotriazole. In addition to the control plasmid (not containing the *gcn5-F221A*), promoter strengths of .07, .32, .68 and .95 were tested (AML31, 32, 34 and 39). cDNA synthesis and quantitative PCR were performed simultaneously using the iScriptTM One-Step RT-PCR Kit with SYBR Green (Bio-Rad). We followed the manufacturer's instructions, with the following modifications: 100 ng of whole cell RNA per 25 µL reaction, an extended, 15 minute reverse transcription time, and a 56°C annealing temperature. For the analysis of yECitrine mRNA levels, BY4741 p415-pPho5-yECitrine, p416-TEF_mutant_-*gcn5-F221A* cell lines were grown in minimal media. In addition to the control strain (no *gcn5-F221A*), promoter strengths of .07, .16, .32 and .68 percent were tested (AML41, 42, 46 and 49). We determined relative RNA concentration by comparing the cycle thresholds to *ALG9*, which has shown to be an ideal housekeeping gene for yeast [53]. Primers 19 and 20 were used to amplify *HIS3*, whereas 21 and 22 were used for *ALG9*. Primers 22 and 23 were used to amplify yECitrine.

Real-time PCR confirmation of microarray findings was conducted on a small scale using whole cell RNA taken from S288C cell lines grown in minimal media (**Fig. S3**). In addition to the control plasmid (no *gcn5-F221A*) and a *gcn5* null strain, a range of promoter strengths were tested (AML31 through 40 **Table S7**). cDNA synthesis was performed using Invitrogen's High Capacity cDNA reverse synthesis kit. For quantitative PCR, we used Roche's SYBR Green Master Mix, following the manufacturer's instructions with an annealing temperature of 58°C. We concentrated on four gene targets; *TKL2*, *SPL2*, *IDH2* and *ZRT1*. Primers 25 and 26 were used for *TKL2*, 27 and 28 for *SPL2*, 29 and 30 for *ZRT1*, and 31 and 32 for *IDH2*. Additionally, *GCN5* mRNA levels were measured with primers 37 and 38, and mRNA extracted from AML31, 32, 34, 35 and 39.

### Gene Expression Microarrays

Global mRNA analysis was conducted using whole cell RNA taken from S288C cell lines grown in minimal media. In addition to the control plasmid (no *gcn5-F221A*) and a *gcn5* null strain, mutant promoter strength 0.32, 0.68 and 0.95 were tested. Cell lines were grown in biological triplicate with a starting optical density of 0.0045 and harvested at a density between 0.4 and 0.5. Whole cell RNA was extracted using the Ambion Ribo-Pure kit for yeast. cRNA synthesis and fragmentation was conducted by the Genome Sequencing and Analysis Facility at the University of Texas using the Ambion MessageAmp Premier kit. Hybridization and scanning was performed by Asuragen in Austin, TX using Affymetrix Yeast 2.0 arrays. Data pre-processing and normalization was performed using the Robust Multichip Average algorithm [54], [55], [56] and Bioconductor's Affy package. Differentially expressed genes were identified using the Linear Models for Microarray Data (LIMMA) package, which resulted in 529 probe sets. Probe sets were matched with *S. cerevisiae* genes using information included in Affymetrix's Expression Console Software, resulting in 504 unique genes. The log_2_ expression data for differentially expressed probe sets are reported in **Table S1** and were deposited to Gene Expression Omnibus under accession number GSE26923.

### Measurements of mutant Gcn5p expression levels

RT-PCR was conducted using whole cell RNA extracted from S288C to determine the level of mutant Gcn5p expression relative to native Gcn5p. Two control strains, wild-type S288C and S288C *gcn5Δ* carried empty vectors. Additionally, S288C with *gcn5-F221A* expressed from varying promoter strengths (.07, .32, .68, .95, and 1.17) were used. Primers were designed such that both wild-type and mutant *GCN5* would be detected. This experiment was carried out as previously described, and average Ct values were normalized with respect to the wild-type sample. Since the sequences are similar between the wild-type and mutant, it was necessary to deduce the expression level. Specifically, we found above that ectopic expression of *gcn5-F221A* does not influence the expression of *GCN5* **(Fig. S4**). Therefore, net changes in the amount of *GCN5/gcn5-F221A* total expression as measured by RT-PCR must be due to changes in the expression of *gcn5-F221A*. This data is shown in **Fig. S2**.

### Growth Analysis for Synthetic Lethal Genes

The impact of the *gcn5* dominant mutant on synthetic lethal genes was assessed using a growth based assay and 22 BY4741 gene knockout strains. As a point of comparison, ten randomly selected, BY4741 null strains served as a control group, and were treated under the same conditions. Synthetic lethals were selected from yGcn5 interaction data, available on yeastgenome.org. Strains AML67 through AML226 (with biological triplicates) were grown in minimal media for 2 days prior to inoculating a honey-comb plate with a starting OD of 0.1. Minimal media lacking uracil was used. Optical density was measured using a Bioscreen C, as previously described. An average growth rate and standard deviation were calculated from the biological replicates. Values (**Table S2**) are reported for each synthetic lethal gene and control strains.

## Supporting Information

Figure S1 **GCN5 complementation assay to determine potential dominant mutants.** Using a BY4741 *gcn5Δ* strain, we expressed two Gcn5p mutants (*gcn5-M193A* and *F221A*) and wild-type GCN5 with varying promoter strengths. Strains were grown in minimal media and growth rate was measured using a Bioscreen C. We compared the mutant growth rates to that of the native yeast. *Gcn5-M193A* was fully functional and no difference in growth rate was observed. However, the *gcn5-F221A* mutant showed no complementation, regardless of promoter strength, making it a good candidate for a dominant mutant. This process was repeated with an S288C *gcn5Δ* strain, in which we expressed two Gcn5p mutants (*gcn5-M193A* and *E173A*) with varying promoter strengths. Again, *gcn5-M193A* was fully functional but *gcn5-E173A* is unable to complement the knockout condition, indicating this mutation interrupts catalytic activity.
(TIF)

Figure S2 **At high promoter strengths, mutant Gcn5p mRNA expression is 8 to 10 times native Gcn5p mRNA levels.** Wild-type and mutant *GCN5* mRNA levels were measured using RT-PCR and whole cell mRNA extracted from S288C wild-type, *gcn5Δ* and *gcn5-F221A* (with 5 different promoter strengths) strains. Average Ct values and standard deviation were calculated from triplicates, and mRNA levels were normalized relative to the wild-type sample. At the lowest promoter strength of .07, mutant *gcn5-F221A* is expressed at levels 2.5 fold higher than wild-type, and at the highest promoter levels of .95 and 1.17, *gcn5-F221A* is expressed at levels 8–10 fold higher than wild-type *GCN5*.
(TIFF)

Figure S3 **RT-PCR of select graded genes confirms microarray findings.** As a follow-up to the *gcn5-F221A* microarray study, 4 graded genes (TKL2, SPL2, ZRT1, IDH2) were selected for higher resolution RT-PCR analysis. RNA was extracted from S288C wild-type cells (▴), S288C *Δgcn5* (▪), S288C *Δgcn5* with p416-TEF_5_-GCN5 (**X**), and S288C with p416-TEF_x_-*gcn5 F221A* (○). Real-time PCR was performed as previously described using primers 25 to 32 for *TKL2*, *SPL2*, *ZRT1* and *IDH2* respectively. Based on the microarray study, *TKL2* was categorized as graded and up-regulated by the dominant mutant with *Δgcn5* displaying false negative behavior. Both trends are reflected in the real time PCR data for *TKL2*. *SPL2*, *ZRT1* and *IDH2* were all categorized as graded and down-regulated by the dominant mutant from the microarray data. Additionally, *Δgcn5* displayed opposite behavior for *ZRT1*. These behaviors are again reflected in the real time PCR data for these three genes. Furthermore, *ZRT1* and *IDH2* show significant gradation at a much higher promoter strength compared to *TKL2* and *SPL2*. This indicates that *TKL2* and *SPL2* are more tightly regulated by Gcn5p.
(TIFF)

Figure S4 **Expression of gcn5-F221A does not influence expression at the native GCN5 promoter.** We sought to determine what, if any, impact, varying expression of mutant Gcn5p had on the expression of native Gcn5p. A p415-pGcn5-yECitrine plasmid, with both a short (•) and long (Δ) *GCN5* promoter, is co-expressed with the p416-TEF_x_-*gcn5-F221A* plasmid collection. In this system, fluorescent protein expression is controlled by the *GCN5* promoter. Regardless of promoter strength, we observed no change in fluorescent expression, which indicates that the p416-TEF_x_-*gcn5-F221A.*
(TIFF)

Figure S5 **Decreased growth rate of S288C caused by cycloheximide treatment of S288C is linked with Gcn5p acetylation activity.** Using an S288C wild-type strain expressing *gcn5-F221A* at varying promoter strengths (○), we measured growth rate in the presence of 0.18 µg/mL cycloheximide. As mutant expression increased, growth rate decreased and approached that of the *gcn5Δ* strain (▪). At low mutant expression levels, growth rate resembled that of the wild-type strain (▴). This demonstrates that the cellular response to cycloheximide is linked with Gcn5p acetylation. A similar impact was observed at the *HIS3* locus (**Fig. 1**), a known Gcn5p gene target.
(TIFF)

Figure S6 **Three low strength TEF promoters constructed using error prone PCR.** Three low strength TEF promoters (0.10±0.01, 0.15±0.01 and 0.22±0.02, measured relative to native TEF promoter) were constructed for this study using error-prone PCR and a fluorescence based screen. Base pair mutations compared to the native TEF promoter are shown in red, underlined text above.
(TIFF)

Figure S7 **Global acetylation at H3K18 is attenuated by expression of mutant gcn5-E173A.** Using immunofluorescence, H3K18 acetylation was assayed globally for strains harboring the *gcn5-E173* mutant expressed with varying promoter strengths (0.32, 0.68, and 0.95), along with wild-type and *gcn5Δ* cells. The primary antibody, raised in rabbit, targets H3K18ac, and the secondary antibody is an anti-rabbit IgG tagged with DyLight 649. All cells were also stained with DAPI to visualize nuclear material. Cells were imaged with both a DAPI and Cy5 filter. The *gcn5-E173A* mutant results in global attenuation of H3K18 acetylation. Using a high strength promoter, acetylation levels are very similar to that of the *gcn5Δ* strain. Average cell intensity quantification, using Metamorph software, confirms that increased *gcn5-E173A* expression decreased acetylation (average cell intensity from left to right: 133050, 89607, 48178, 37252, 32128).
(TIF)

Table S1 **Differentially Expressed Genes and Characterization from Global Microarray Study.** From our global microarray study, we identified 504 unique genes that were differentially expressed and statistically significant between sample sets. Here we have catalogued and characterized each of those genes and the phenomena we observed with regard to the *gcn5-F221A* dominant mutant. Gene IDs were matched from Affymetrix probe sets as previously described (**Methods**) and gene names are listed when available. Log_2_ expression change is calculated with respect to the control. Tef3, 11 and 5 promoters have strengths of 0.32, 0.68 and 0.95 respectively compared to a native TEF promoter. Catalytically associated genes are denoted with a ‘CA’, and ‘U’ for up-regulated and ‘D’ for down-regulated genes. ‘NCA’, ‘FN’, and ‘OPP’ correspond to ‘non-catalytically associated, ‘false negative’ and opposite respectively. Some genes are listed in multiple categories. ‘Uncat’ denotes a gene that cannot be categorized based on the criteria previously outlined.
(XLS)

Table S2 **Synthetic lethal gene knockouts are impacted by gcn5-F221A dominant mutant.** Growth rates for twenty-two *gcn5Δ* synthetic lethal gene knockouts were determined under varying dominant mutant expression levels using a Bioscreen C and compared to 10 randomly selected, BY4741 null strains serving as a control group. Average growth rates and standard deviations were calculated from biological triplicates. While some of the synthetic lethal strains show a graded response to the *gcn5-F221A* mutant, none of the control strains are impacted by the mutant.
(DOC)

Table S3 **Putative GCN5-Dependant Growth Inhibitors were tested for an impact with GCN5 mutant.** Previous studies have identified the following compounds and concentrations which inhibited growth of a *Δgcn5* strain compared to wild-type yeast. We tested the growth of S288C strains expressing *gcn5-F221A* in the presence of these compounds in liquid media, as described in the **Materials and Methods**.
(DOC)

Table S4 **Host Strains.** Both *E. coli* and *S. cerevisiae* strains were used in this study, as outlined in Materials and Methods. Each strain, its genotype and the source from which we obtained the strain are listed above.
(XLS)

Table S5 **Plasmid Carrying Strains.** One hundred seventy-six strains were used in this study, as outlined in Materials and Methods. Each strain has been assigned a number (AML1 through AML262), corresponding to a specific genotype, and plasmid(s) specific to the strain.
(XLS)

Table S6 **Primer sequences.** Primers used throughout this study are listed here.
(DOC)

Table S7 **Reaction conditions for error-prone PCR.** Three new low strength mutant TEF promoters were developed for this study (**Fig. S2**) using error-prone PCR and a fluorescence based screen. The error-prone PCR conditions (shown above) resulted in a mutation rate between 4 and 13.5 per kilobase.
(DOC)

Discussion S1 (DOC)

Discussion S2 (DOC)

Discussion S3 (DOC)

Discussion S4 (DOC)
