# Supplementary figures and images for: Linking Yeast Gcn5p Catalytic Function and Gene Regulation Using a Quantitative, Graded Dominant Mutant Approach
Source: PLoS One. 2012 Apr 27;7(4):e36193. doi: 10.1371/journal.pone.0036193 (PMC3338614; doi:10.1371/journal.pone.0036193)

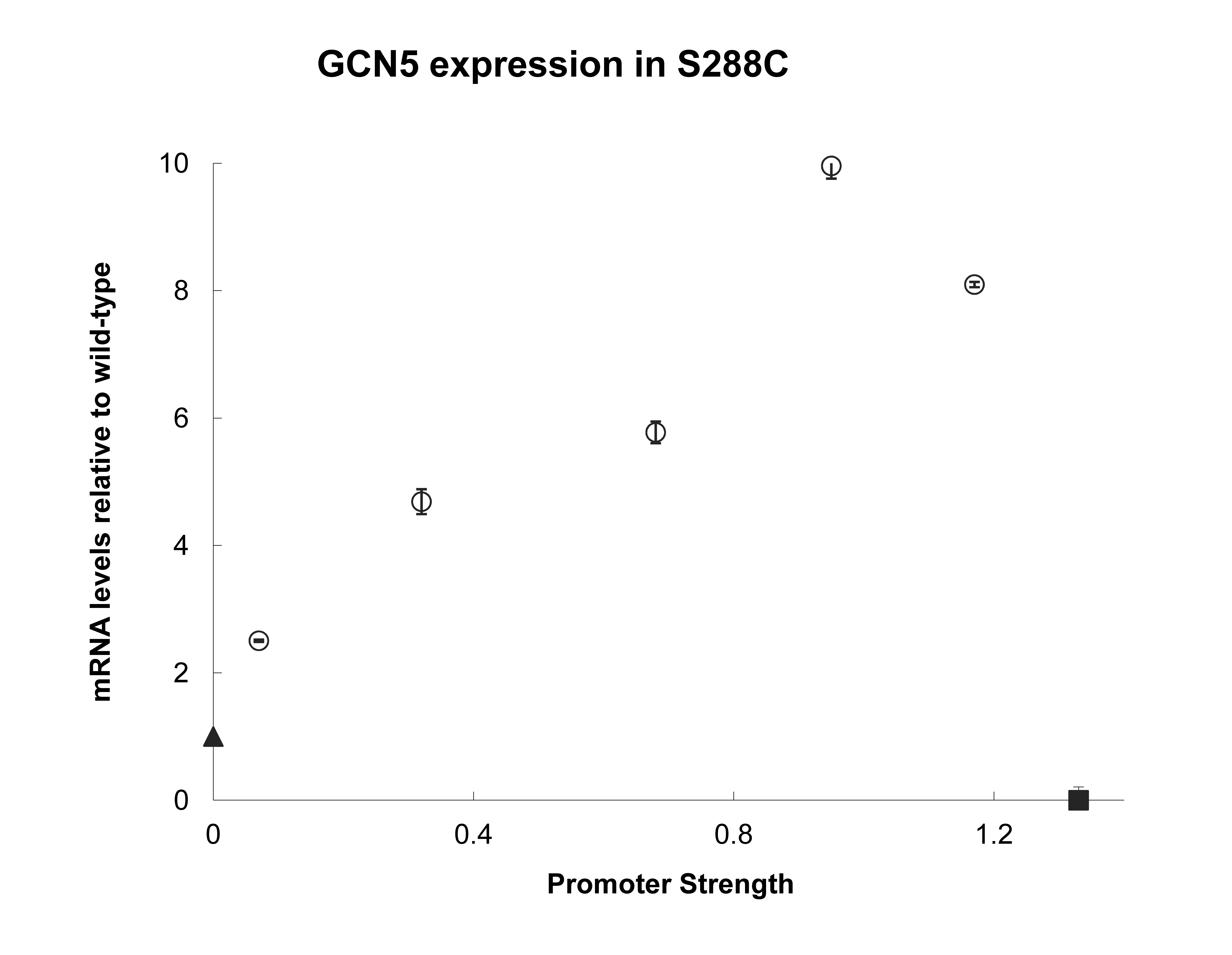

Supplement: Figure S2 — At high promoter strengths, mutant Gcn5p mRNA expression is 8 to 10 times native Gcn5p mRNA levels. Wild-type and mutant GCN5 mRNA levels were measured using RT-PCR and whole cell mRNA extracted from S288C wild-type, gcn5Δ and gcn5-F221A (with 5 different promoter strengths) strains. Average Ct values and standard deviation were calculated from triplicates, and mRNA levels were normalized relative to the wild-type sample. At the lowest promoter strength of .07, mutant gcn5-F221A is expressed at levels 2.5 fold higher than wild-type, and at the highest promoter levels of .95 and 1.17, gcn5-F221A is expressed at levels 8–10 fold higher than wild-type GCN5. (TIFF) [file pone.0036193.s002.tiff]

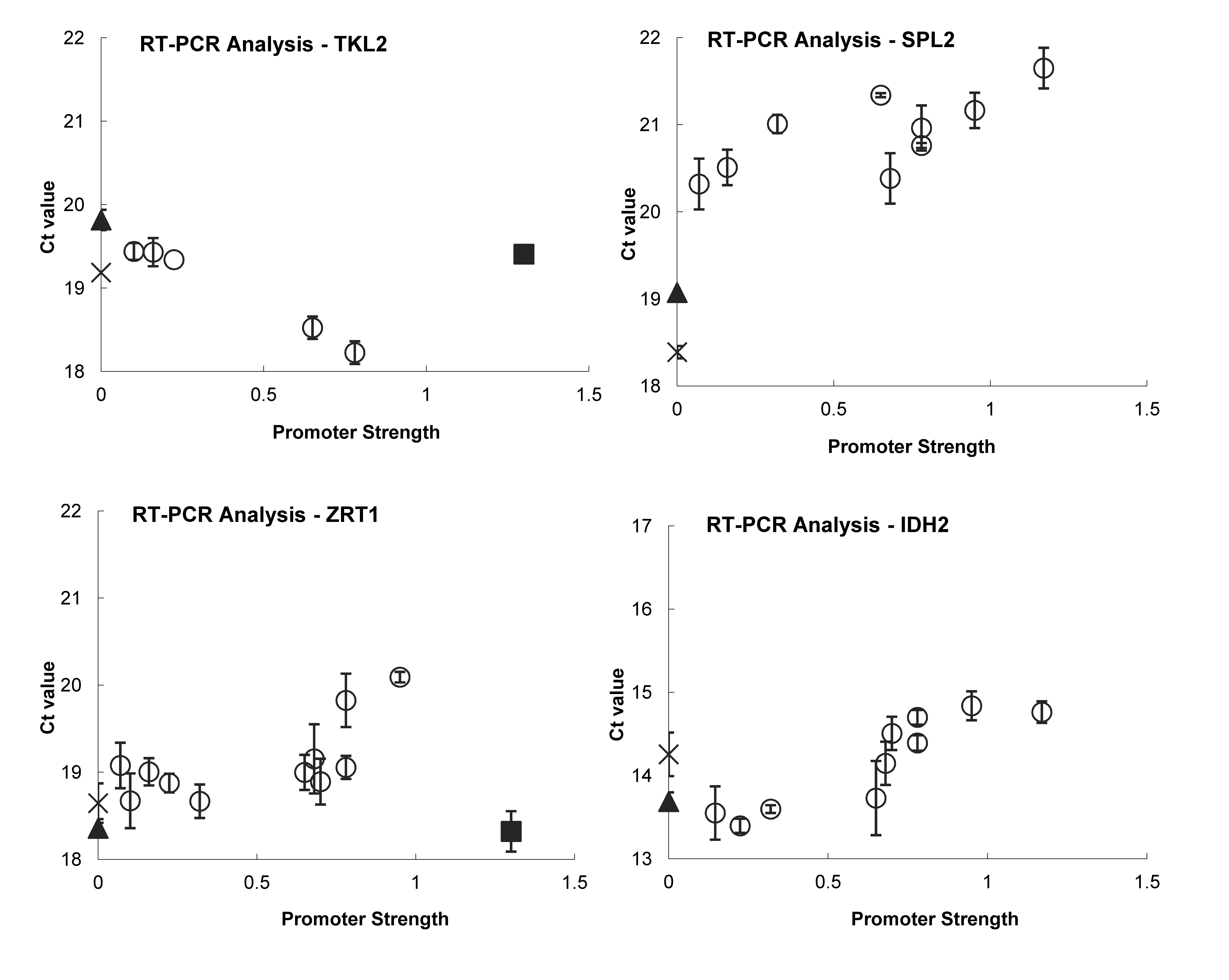

Supplement: Figure S3 — RT-PCR of select graded genes confirms microarray findings. As a follow-up to the gcn5-F221A microarray study, 4 graded genes (TKL2, SPL2, ZRT1, IDH2) were selected for higher resolution RT-PCR analysis. RNA was extracted from S288C wild-type cells (▴), S288C Δgcn5 (▪), S288C Δgcn5 with p416-TEF5-GCN5 (X), and S288C with p416-TEFx-gcn5 F221A (○). Real-time PCR was performed as previously described using primers 25 to 32 for TKL2, SPL2, ZRT1 and IDH2 respectively. Based on the microarray study, TKL2 was categorized as graded and up-regulated by the dominant mutant with Δgcn5 displaying false negative behavior. Both trends are reflected in the real time PCR data for TKL2. SPL2, ZRT1 and IDH2 were all categorized as graded and down-regulated by the dominant mutant from the microarray data. Additionally, Δgcn5 displayed opposite behavior for ZRT1. These behaviors are again reflected in the real time PCR data for these three genes. Furthermore, ZRT1 and IDH2 show significant gradation at a much higher promoter strength compared to TKL2 and SPL2. This indicates that TKL2 and SPL2 are more tightly regulated by Gcn5p. (TIFF) [file pone.0036193.s003.tiff]

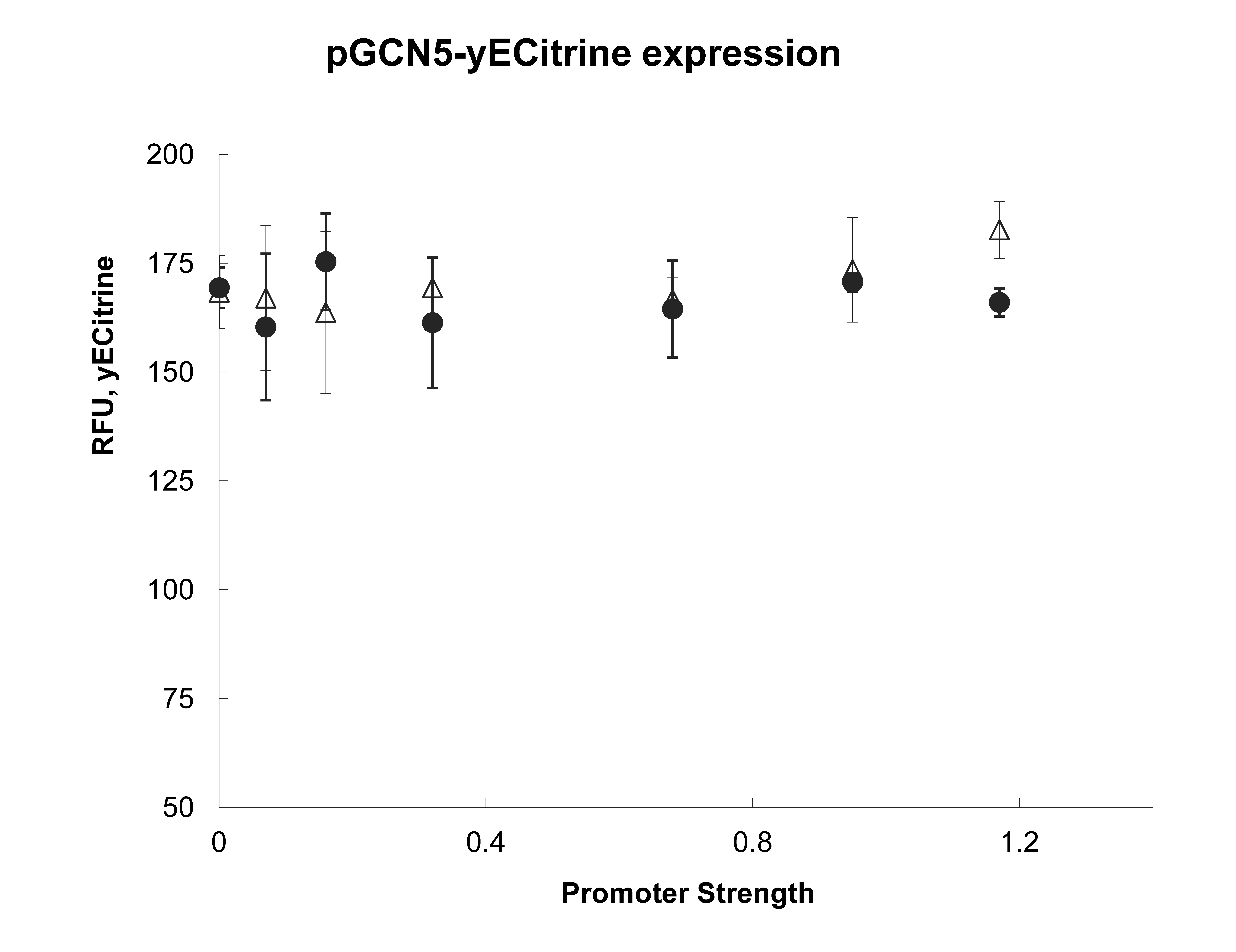

Supplement: Figure S4 — Expression of gcn5-F221A does not influence expression at the native GCN5 promoter. We sought to determine what, if any, impact, varying expression of mutant Gcn5p had on the expression of native Gcn5p. A p415-pGcn5-yECitrine plasmid, with both a short (•) and long (Δ) GCN5 promoter, is co-expressed with the p416-TEFx-gcn5-F221A plasmid collection. In this system, fluorescent protein expression is controlled by the GCN5 promoter. Regardless of promoter strength, we observed no change in fluorescent expression, which indicates that the p416-TEFx-gcn5-F221A. (TIFF) [file pone.0036193.s004.tiff]

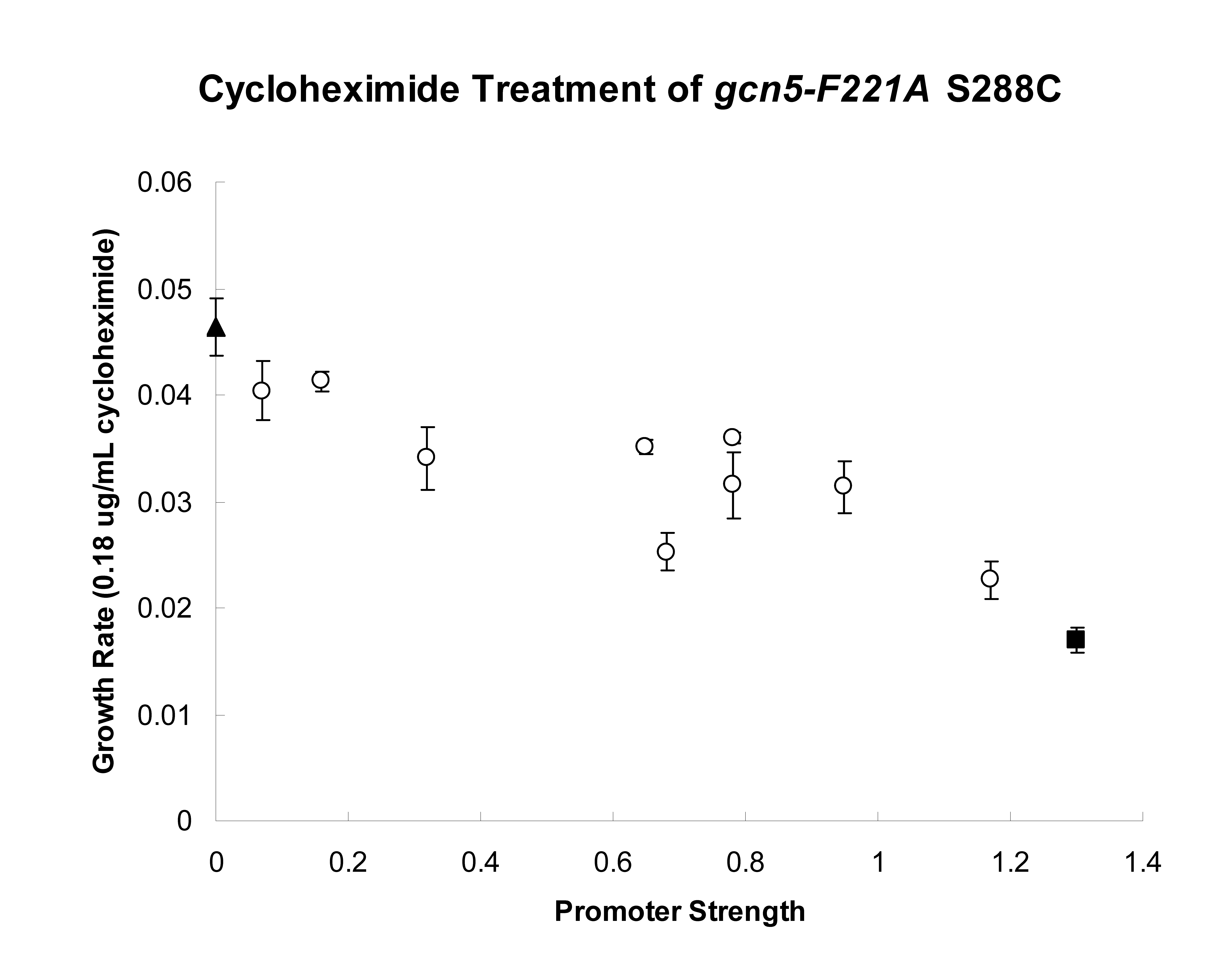

Supplement: Figure S5 — Decreased growth rate of S288C caused by cycloheximide treatment of S288C is linked with Gcn5p acetylation activity. Using an S288C wild-type strain expressing gcn5-F221A at varying promoter strengths (○), we measured growth rate in the presence of 0.18 µg/mL cycloheximide. As mutant expression increased, growth rate decreased and approached that of the gcn5Δ strain (▪). At low mutant expression levels, growth rate resembled that of the wild-type strain (▴). This demonstrates that the cellular response to cycloheximide is linked with Gcn5p acetylation. A similar impact was observed at the HIS3 locus ( Fig. 1 ), a known Gcn5p gene target. (TIFF) [file pone.0036193.s005.tiff]

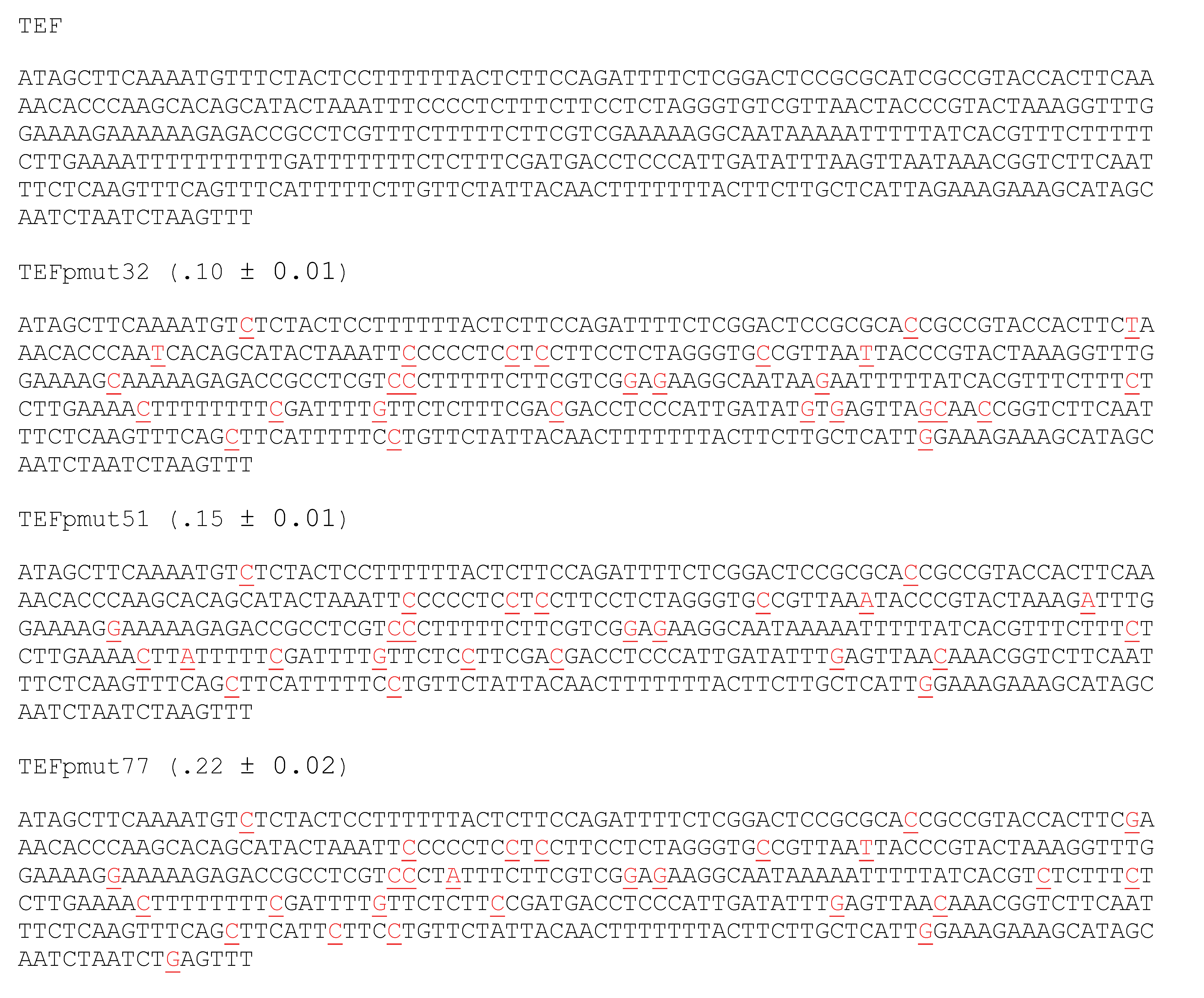

Supplement: Figure S6 — Three low strength TEF promoters constructed using error prone PCR. Three low strength TEF promoters (0.10±0.01, 0.15±0.01 and 0.22±0.02, measured relative to native TEF promoter) were constructed for this study using error-prone PCR and a fluorescence based screen. Base pair mutations compared to the native TEF promoter are shown in red, underlined text above. (TIFF) [file pone.0036193.s006.tiff]

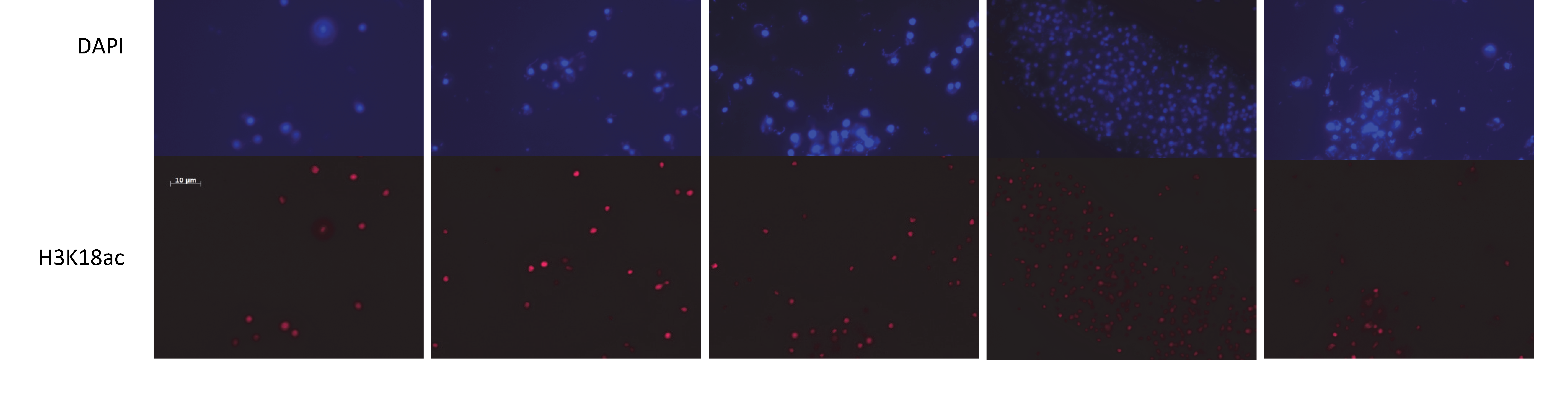

Supplement: Figure S7 — Global acetylation at H3K18 is attenuated by expression of mutant gcn5-E173A. Using immunofluorescence, H3K18 acetylation was assayed globally for strains harboring the gcn5-E173 mutant expressed with varying promoter strengths (0.32, 0.68, and 0.95), along with wild-type and gcn5Δ cells. The primary antibody, raised in rabbit, targets H3K18ac, and the secondary antibody is an anti-rabbit IgG tagged with DyLight 649. All cells were also stained with DAPI to visualize nuclear material. Cells were imaged with both a DAPI and Cy5 filter. The gcn5-E173A mutant results in global attenuation of H3K18 acetylation. Using a high strength promoter, acetylation levels are very similar to that of the gcn5Δ strain. Average cell intensity quantification, using Metamorph software, confirms that increased gcn5-E173A expression decreased acetylation (average cell intensity from left to right: 133050, 89607, 48178, 37252, 32128). (TIF) [file pone.0036193.s007.tif]
