# Supplementary material for: Linking Yeast Gcn5p Catalytic Function and Gene Regulation Using a Quantitative, Graded Dominant Mutant Approach
Source: PLoS One. 2012 Apr 27;7(4):e36193. doi: 10.1371/journal.pone.0036193 (PMC3338614; doi:10.1371/journal.pone.0036193)
Supplement: Table S2 — Synthetic lethal gene knockouts are impacted by gcn5-F221A dominant mutant. Growth rates for twenty-two gcn5Δ synthetic lethal gene knockouts were determined under varying dominant mutant expression levels using a Bioscreen C and compared to 10 randomly selected, BY4741 null strains serving as a control group. Average growth rates and standard deviations were calculated from biological triplicates. While some of the synthetic lethal strains show a graded response to the gcn5-F221A mutant, none of the control strains are impacted by the mutant. (DOC) [file pone.0036193.s009.doc]

Table S2

| ***gcn5* synthetic lethal** | **Control growth rate (1/h)** | **.16 growth rate (1/h)** | **.32 growth rate (1/h)** | **.68 growth rate (1/h)** | **.95 growth rate (1/h)** |
| --- | --- | --- | --- | --- | --- |
| *ccr4* | .225 ± .003 | .218 ± .007 | .192 ± .000 | .180 ± .003 | .168 ± .001 |
| *eaf7* | .252 ± .003 | .225 ± .001 | .232 ± .002 | .225 ± .001 | .208 ± .003 |
| *elp3* | .229 ± .001 | .220 ± .002 | .212 ± .002 | .208 ± .001 | .208 ± .002 |
| *hhf2* | .249 ± .003 | .234 ± .004 | .222 ± .001 | .209 ± .002 | .206 ± .002 |
| *hht2* | .253 ± .006 | .227 ± .022 | .238 ± .004 | .209 ± .006 | .221 ± .009 |
| *hsl1* | .260 ± .006 | .250 ± .004 | .244 ± .002 | .228 ± .009 | .222 ± .013 |
| *hsl7* | .244 ± .001 | .232 ± .004 | .243 ± .005 | .216 ± .007 | .197 ± .003 |
| *iki3* | .225 ± .002 | .217 ± .002 | .219 ± .003 | .208 ± .002 | .206 ± .003 |
| *leu2* | .255 ± .004 | .237 ± .004 | .232 ± .002 | .227 ± .002 | .219 ± .001 |
| *mot2* | .271 ± .008 | .248 ± .005 | .258 ± .006 | .234 ± .003 | .230 ± .003 |
| *nam2* | .233 ± .001 | .228 ± .003 | .235 ± .000 | .217 ± .005 | .219 ± .000 |
| *not5* | .259 ± .002 | .246 ± .003 | .244 ± .001 | .230 ± .001 | .221 ± .002 |
| *paa1* | .245 ± .005 | .236 ± .004 | .195 ± .004 | .188 ± .005 | .197 ± .010 |
| *pap2* | .263 ± .005 | .250 ± .002 | .246 ± .002 | .236 ± .001 | .232 ± .006 |
| *pho23* | .231 ± .003 | .228 ± .001 | .218 ± .004 | .219 ± .004 | .210 ± .003 |
| *rad6* | .193 ± .008 | .180 ± .002 | .176 ± .005 | .163 ± .004 | .159 ± .002 |
| *rpd3* | .260 ± .003 | .247 ± .005 | .240 ± .002 | .232 ± .002 | .222 ± .003 |
| *rtt109* | .204 ± .003 | .167 ± .010 | .154 ± .007 | .131 ± .006 | .131 ± .004 |
| *rsc2* | .209 ± .013 | .194 ± .001 | .194 ± .004 | .167 ± .006 | .158 ± .000 |
| *sin3* | .195 ± .001 | .183 ± .002 | .186 ± .008 | .162 ± .022 | .173 ± .005 |
| *snf2* | .176 ± .003 | .161 ± .012 | .139 ± .020 | .146 ± .002 | .152 ± .019 |
| *spt20* | 0.149 ± .001 | 0.144 ± .002 | 0.151 ± .002 | 0.142 ± .002 | 0.140 ± .004 |
| **Random BY4741 null strains** | **Control growth rate (1/h)** | **.16 growth rate (1/h)** | **.32 growth rate (1/h)** | **.68 growth rate (1/h)** | **.95 growth rate (1/h)** |
| *cad1* | .155 ± .022 | .128 ± .070 | .190 ± .018 | .163 ± .028 | .175 ± .024 |
| *hpa2* | .124 ± .006 | .161 ± .024 | .139 ± .006 | .138 ± .024 | .132 ± .007 |
| *lsb3* | .136 ± .039 | .131 ± .042 | .130 ± .007 | .131 ± .006 | .132 ± .003 |
| *nma2* | .163 ± .031 | .141 ± .015 | .147 ± .022 | .114 ± .014 | .160 ± .007 |
| *nup170* | .125 ± .014 | .135 ± .023 | .151 ± .023 | .110 ± .014 | .159 ± .020 |
| *rpl20b* | .120 ± .034 | .113 ± .019 | .128 ± .063 | .125 ± .011 | .152 ± .018 |
| *vps28* | .147 ± .017 | .138 ± .023 | 0.139 ± .018 | .145 ± .002 | 0.126 ± .043 |
| YBR287W | .154 ± .021 | .172 ± .022 | .144 ± .033 | .144 ± .002 | .138 ± .034 |
| YML131W | .138 ± .009 | .120 ± .007 | .144 ± .031 | .166 ± .016 | .126 ± .022 |
| YNL234W | .141 ± .019 | 0.164 ± .017 | 0.165 ± .040 | 0.154 ± .020 | 0.154 ± .039 |
