# Supplementary material for: Linking Yeast Gcn5p Catalytic Function and Gene Regulation Using a Quantitative, Graded Dominant Mutant Approach
Source: PLoS One. 2012 Apr 27;7(4):e36193. doi: 10.1371/journal.pone.0036193 (PMC3338614; doi:10.1371/journal.pone.0036193)
Supplement: Table S3 — Putative GCN5-Dependant Growth Inhibitors were tested for an impact with GCN5 mutant. Previous studies have identified the following compounds and concentrations which inhibited growth of a Δgcn5 strain compared to wild-type yeast. We tested the growth of S288C strains expressing gcn5-F221A in the presence of these compounds in liquid media, as described in the Materials and Methods . (DOC) [file pone.0036193.s010.doc]

Table S3

| Putative *GCN5*-Dependent Growth Inhibitor | Test Culture Concentration | Previous Study Type | Previous Media Type |
| --- | --- | --- | --- |
| Ethanol | 6% w/v | High throughput | Solid |
| Cycloheximide | 0.18μg/mL | High throughput | Solid |
| Sulfanilamide | 200μg/mL | High throughput | Liquid |
| 5-Fluorouracil | 15μg/mL | High throughput | Solid |
| KCl | 1M | Individual | Solid |
| CaCl_2_ | 0.25M | Individual | Solid |
| MnCl_2_ | 4mM | Individual | Solid |
| MnCl_2_ | 8mM | Individual | Solid |
| MnCl_2_ | 40mM | Individual | Solid |
| MnCl_2_ | 200mM | Individual | Solid |
