# Supplementary material for: Linking Yeast Gcn5p Catalytic Function and Gene Regulation Using a Quantitative, Graded Dominant Mutant Approach
Source: PLoS One. 2012 Apr 27;7(4):e36193. doi: 10.1371/journal.pone.0036193 (PMC3338614; doi:10.1371/journal.pone.0036193)
Supplement: Table S6 — Primer sequences. Primers used throughout this study are listed here. (DOC) [file pone.0036193.s013.doc]

**Table S6**

| **Primer** | **Sequence** |
| --- | --- |
| 1 | AGTCTTCAGTTAACTCAGGTTCGTATTCTACATTAGATGGGCGCGCCAGATCTGTTTAGC |
| 2 | CGAAAGGAATAGTAGCGGAAAAGCTTCTTCTACGCATTACGTTTTCGACACTGGATGGCG |
| 3 | GATTGGTAAGGGAAGACCGTGAGCCGCCCAAAAGTCTTCAGTTAACTCAGGTTCGTATTC |
| 4 | ACATCGTCTCGCCGTACTAAACATTTATTTCTTCTTCGAAAGGAATAGTAGCGGAAAAGC |
| 5 | CGGGGATTCCCAATACGAGGTCGCC |
| 6 | CCTCAATTGATCACATCGTCTCGCCGTAC |
| 7 | CAGAAACTTCTCGACAGACGTCGCGG |
| 8 | GTAGGGCGTAATGATGTTTGCTTGTCAAC |
| 9 | CTAGTCTAGAAAAATGGTCACAAAACATCAGATTGAAGAGGATC |
| 10 | CTAGCGGTCGACTTAATCAATAAGGTGAGAATATTCAGGTATTTCTTTTACTTTATTATT |
| 11 | GCAGATAATTACGCTATTGGATACGCTAAAAAGCAAGGCTTCACTAAAG |
| 12 | CTTTAGTGAAGCCTTGCTTTTTAGCGTATCCAATAGCGTAATTATCTGC |
| 13 | CGGTTATGGTGCGCATCTAGCGAATCACTTAAAAGACTATGTTAG |
| 14 | CTAACATAGTCTTTTAAGTGATTCGCTAGATGCGCACCATAACCG |
| 15 | GCTAAAAGGCCTTAGGTCTAGAGATCTGTTTAGCTTGCCTCG |
| 16 | ATTACTGATATCATTAAGGGTTCTCGAGAGCTCGTTTTCG |
| 17 | GCTAGCGAGCTCTAAATACAATGTTCCTTGGTTATCCCATCGCC |
| 18 | GCTCTAGATGGTAATCTCGAATTTGCTTGCTCTATTTGTTGT |
| 19 | ACGACCATCACACCACTGAAGACT |
| 20 | CCAAAGGCGCAAATCCTGATCCAA |
| 21 | ATCGTGAAATTGCAGGCAGCTTGG |
| 22 | CATGGCAACGGCAGAAGGCAATAA |
| 23 | TTCTGTCTCCGGTGAAGGTGAA |
| 24 | TAAGGTTGGCCATGGAACTGGCAA |
| 25 | CCAACTTTGCCGCCACTTAT |
| 26 | TTGTAAGCAACCATCCCCTACA |
| 27 | ACATCGCAGTCACAATCTCTCAGT |
| 28 | CATGGGCGAGCTTGCTTAAA |
| 29 | AGTAGCGTCTGGGTTGAAAGAAAGTA |
| 30 | CAGTGTTTCTCACAACAGTGTCTTTAAT |
| 31 | TGACATTGAGAAAAACATTTGGGTTA |
| 32 | TCAACGTTTTCGTAAGTGGTCTTAA |
| 33 | CCTCACTAAAGGGAACAAAAGCTG |
| 34 | CAGTGAATAATTCTTCACCTTTAGACATTTT |
| 35 | TGTTGTGTGGAATTGTGAGC |
| 36 | TAGCATCACCTTCACCTTCAC |
| 37 | CGGCTGGACTCCCGAGAT |
| 38 | TTGTAGCTCTGTGAGTATATTCTGTATTGC |
| 39 | CGATAAGAGAGAATTCGCAGCAATTGTTTTCTGTGCCATCA |
| 40 | TGATGGCACAGAAAACAATTGCTGCGAATTCTCTCTTATCG |
| 41 | CACACACGAGCTCAGAGCAAAGACAAAAAAAATAAGACA |
| 42 | CTAGTCTAGACTAATGTAGAATACGAACCTGAGTT |
| 43 | CACACACGAGCTCTCTTAAACACTTATGGGCAGC |
