# Supplementary material for: Linking Yeast Gcn5p Catalytic Function and Gene Regulation Using a Quantitative, Graded Dominant Mutant Approach
Source: PLoS One. 2012 Apr 27;7(4):e36193. doi: 10.1371/journal.pone.0036193 (PMC3338614; doi:10.1371/journal.pone.0036193)
Supplement: Table S7 — Reaction conditions for error-prone PCR. Three new low strength mutant TEF promoters were developed for this study (Fig. S2) using error-prone PCR and a fluorescence based screen. The error-prone PCR conditions (shown above) resulted in a mutation rate between 4 and 13.5 per kilobase. (DOC) [file pone.0036193.s014.doc]

**Table S7**

| Reaction | Library | Plasmid Template (ng) | Mutation Rate (1/kb) |
| --- | --- | --- | --- |
| 1 | Low | 30.18 | 7.5 +/- 3.5 |
| 2 |  | 9.62 |  |
| 3 |  | 3.07 |  |
| 4 | High | 0.98 | 9.2 +/- 4.3 |
| 5 |  | 0.31 |  |
| 6 |  | 0.10 |  |
