# Supplementary material for: Linking Yeast Gcn5p Catalytic Function and Gene Regulation Using a Quantitative, Graded Dominant Mutant Approach
Source: PLoS One. 2012 Apr 27;7(4):e36193. doi: 10.1371/journal.pone.0036193 (PMC3338614; doi:10.1371/journal.pone.0036193)
Supplement: Discussion S1 — (DOC) [file pone.0036193.s015.doc]

***gcn5-E173A*, a second dominant mutant**

We sought to demonstrate that the graded dominant mutant approach is generic, and that the loss of catalytic activity in *gcn5-F221A* was not unique to this particular mutation. We selected a third *gcn5* mutation, E173A. In a previous study, a three amino acid stretch (FAE 171-173) was mutated to alanine, resulting in a loss of catalytic activity [[1]](#_ENREF_14). This mutant *gcn5* protein, along with *gcn5-M193A,* was cloned into yeast expression vectors with varying promoter strengths and a G418 resistance gene. Plasmids were transformed into the S288C *Δgcn5* strain. *gcn5-M193A* was selected as a control experiment because previous results indicate this mutant is fully active. One again, we performed a complementation assay and tested for growth rate in the presence of 3-aminotriazole. We observed that the *gcn5*-*E173A* mutant is unable to complement *gcn5Δ* and behaves similar to the knockout strain. As expected, the *M193A* mutants exhibited the same growth rates as the control strain regardless of promoter strength (**Fig. S1**). Again, we used PISA to determine that the E173A mutation results in no change to Gcn5p Gibbs Free energy.

As further validation that *gcn5-E173A* functions as a graded dominant mutant able to competitively inhibit native Gcn5p, we assayed for global histone acetylation. H3K18 is a common acetylation target for Gcn5p and acetylation of this site can be quantified using immunofluorescence. Strains harboring the *gcn5-E173A* mutant expressed with varying promoter strengths (0.32, 0.68, and 0.95) were assayed, along with wild-type and *gcn5Δ* cells. The primary antibody, raised in rabbit, targets H3K18ac, and the secondary antibody is an anti-rabbit IgG tagged with DyLight 649. All cells were also stained with DAPI to visualize nuclear material. Cells were imaged with both a DAPI and Cy5 filter. As shown in **Fig. S7**, the *gcn5-E173A* mutant results in global attenuation of H3K18 acetylation. Using a high strength promoter, acetylation levels are very similar to that of the *gcn5Δ* strain. Average cell intensity was quantified using Metamorph software. For the wild-type, *gcn5-E173A* (0.32, 0.68, and 0.95) and *gcn5Δ* cells, average cell intensities are 133050, 89607, 48178, 37252 and 32128 respectively. These results indicate that like *gcn5-F221A*, this second, graded dominant mutant is able to competitively inhibit Gcn5p and directly interfere with acetylation activity. Moreover, these results suggest that this same analysis could have been conducted using this mutant in place of *gcn5-F221A*, thus illustrating the only two requirements of this approach: a dominant mutation and a promoter library.

**References**

1. Wang L, Liu L, Berger SL (1998) Critical residues for histone acetylation by Gcn5, functioning in Ada and SAGA complexes, are also required for transcriptional function in vivo. Genes Dev 12: 640-653.
