# Supplementary material for: Linking Yeast Gcn5p Catalytic Function and Gene Regulation Using a Quantitative, Graded Dominant Mutant Approach
Source: PLoS One. 2012 Apr 27;7(4):e36193. doi: 10.1371/journal.pone.0036193 (PMC3338614; doi:10.1371/journal.pone.0036193)
Supplement: Discussion S2 — (DOC) [file pone.0036193.s016.doc]

**Real-Time PCR for select graded genes**

Our global microarray study identified 288 genes whose expression levels were impacted by the *gcn5-F221A* dominant mutant. We selected 4 of these graded genes; *TKL2*, *SPL2*, *IDH2* and *ZRT1*, and quantified the impact of dominant mutant expression on mRNA levels using real-time PCR. These tests confirmed the results of the microarray study, while adding better resolution by including additional promoter strengths. These results are depicted in **Fig. S3**. As an additional complementation control, we included a p416-TEF5-*GCN5* wild-type plasmid transformed into *gcn5Δ*. The cycle threshold values for this complementation control match very closely with that of the wild-type yeast, indicating that the observed differences in gene expression in both this study and the microarray study are not an artifact of replicative plasmids expressing high levels of Gcn*5*p.
