# Supplementary material for: Linking Yeast Gcn5p Catalytic Function and Gene Regulation Using a Quantitative, Graded Dominant Mutant Approach
Source: PLoS One. 2012 Apr 27;7(4):e36193. doi: 10.1371/journal.pone.0036193 (PMC3338614; doi:10.1371/journal.pone.0036193)
Supplement: Discussion S3 — (DOC) [file pone.0036193.s017.doc]

**Chromatin DB systems analysis of microarray data**

We sought to determine which covalent chromatin modifications, if any, were enriched or depleted under the various conditions explored in our microarray experiment. Using Chromatin DB [[1]](#_ENREF_16), which utilizes the Bonferroni Correction, we examined those genes (1) differentially expressed between the wild-type and knockout, (2) graded by *gcn5-F221A*, (3) differentially expressed between the wild-type and knockout, but not graded (non-catalytically associated), (4) graded but not differentially expressed between the wild-type and knockout (False negatives), and (5) genes with knockout expression levels opposite the expression levels in the presence of the *gcn5-F221A* mutant (opposites). We have catalogued those statistically significant covalent modifications for which each data set is enriched and depleted with corrected p values in parentheses.

1. Differentially expressed (DE) genes between wild-type & knockout

No significant enrichment or depletion of chromatin

DE genes between wild-type & knockout, up-regulated in knockout

Depletion: H4Nterm ac (<10^-3^)

DE genes between wild-type & knockout, down-regulated in knockout

Enrichment: H3K4me_2_ (<10^-4^)

Depletion: H3 occupancy (<10^-4^), H4 occupancy (<10^-3^)

DE genes between wild-type & knockout, no grading observed

No significant enrichment or depletion of chromatin

1. Graded genes compared to wild-type

No significant enrichment or depletion of chromatin

Graded up compared to wild-type

Depletion: H3K18ac (<10^-3^), H3K14ac (<10^-3^)

Graded up, early

No significant enrichment or depletion of chromatin

Graded up, late

Depletion: H2AK7ac (<10^-4^), H2BK11ac (<10^-4^), H2BK16ac (<10^-3^), H3K14ac (<10^-4^), H3K18ac (<10^-4^), H3K23ac (<10^-4^)

Graded down compared to wild-type

Depletion: H4 occupancy (<10^-3^)

Graded down, early

No significant enrichment or depletion of chromatin

Graded down, late

Depletion: H4 occupancy (<10^-3^)

Graded up compared to wild-type, no change in knockout

No significant enrichment or depletion of chromatin

Graded down compared to wild-type, no change in knockout

No significant enrichment or depletion of chromatin

1. Non-catalytically associated genes (differentially expressed in knockout, no gradation)

Depletion: H2AZ occupancy (<10^-3^)

1. False negative genes

Depletion: H2BK11ac (<10^-3^), H2BK16ac (<10^-3^), H3K18ac (<10^-4^), H3K14ac (<10^-4^), H3K23ac (<10^-4^),

1. Opposite genes

Depletion: H4K16ac (<10^-3^), H2AZ occupancy (<10^-3^)

**References**

1. O'Connor TR, Wyrick JJ (2007) ChromatinDB: a database of genome-wide histone modification patterns for Saccharomyces cerevisiae. Bioinformatics 23: 1828-1830.
