# Supplementary material for: Linking Yeast Gcn5p Catalytic Function and Gene Regulation Using a Quantitative, Graded Dominant Mutant Approach
Source: PLoS One. 2012 Apr 27;7(4):e36193. doi: 10.1371/journal.pone.0036193 (PMC3338614; doi:10.1371/journal.pone.0036193)
Supplement: Discussion S4 — (DOC) [file pone.0036193.s018.doc]

**Cytoscape BiNGO 2.44 systems analysis of microarray data**

From our microarray study, we sought to determine which cellular processes, if any, were related to the observed cellular phenomena. Using Cytoscape BiNGO version 2.44 [[1]](#_ENREF_17), we examined the over represented cellular processes (**ORCP**) and underrepresented cellular processes (**URCP**) for subsets of genes. We examined those genes (1) differentially expressed between the wild-type and knockout, (2) catalytically associated, (3) differentially expressed between the wild-type and knockout, but not catalytically associated, (4) catalytically associated but not differentially expressed between the wild-type and knockout (False negatives), and (5) genes with knockout expression levels opposite the expression levels in the presence of the *gcn5-F221A* mutant (opposites). Statistically significant ORCPs and URCPs are reported below, with corrected p values calculated for false discovery rates in parentheses, except in the case of the false negatives and opposites for which there were no statistically significant ORCPs or URCPs.

1. Differentially expressed (DE) genes between wild-type & knockout

ORCP: cell wall (2.10e-4), conjugation (4.83e-2), extra-cellular region (4.94e-4), plasma membrane (1.57e-4)

URCP: cytoplasm (9.03e-10), DNA metabolic process (6.93e-4), triplet codon-amino acid adaptor activity (3.89e-5), endomembrane system (2.31e-2), ribosome (8.88e-4), mitochondrial envelope (3.55e-2), response to stress (1.74e-2), chromosome segregation (3.55e-2), golgi apparatus (3.55e-2), protein complex biogenesis (3.55e-2), translation (2.34e-2), structural molecule activity (3.55e-2)

DE genes between wild-type & knockout, up-regulated in knockout

ORCP: cell wall (1.80e-3)

URCP: cytoplasm (5.29e-3), DNA metabolic process (5.29e-3), triplet codon-amino acid adaptor activity (1.42e-2), translation (3.13e-2), nucleolus (2.40e-2), nucleus (8.55e-3), ribosome biogenesis (5.29e-3), RNA binding (6.51e-4), RNA metabolic process (3.49e-4)

DE genes between wild-type & knockout, down-regulated in knockout

ORCP: nucleolus (4.39e-5), ribosome biogenesis (3.05e-4), conjugation (3.14e-4), plasma membrane (3.60e-3), extra-cellular region (2.02e-2), RNA metabolic process (4.05e-2)

URCP: cytoplasm (2.65e-7), response to stress (1.04e-2), triplet codon-amino acid adaptor activity (2.17e-2), protein binding (2.17e-2), mitochondrial envelope (2.17e-2), structural molecule activity (2.17e-2), ribosome (2.17e-2), golgi apparatus (3.70e-2), protein complex biogenesis (3.70e-2)

DE genes between wild-type & knockout, no grading observed

URCP: RNA metabolic process (3.32e-2), RNA binding (3.32e-2)

2. Graded genes compared to wild-type

ORCP: oxidoreductase activity (3.00e-2)

URCP: triplet codon-amino acid adaptor activity (2.71e-5), cytoplasm (3.27e-3), translation (1.42e-5), ribosome (8.70e-4), structural molecule activity (2.25e-2)

Graded up compared to wild-type

ORCP: Oxidoreductase activity (8.86e-3), cellular protein catabolic process (3.16e-2)

URCP: RNA binding (2.28e-2), triplet codon-amino acid adaptor activity (3.11e-3), Ribosome biogenesis (3.11e-3), RNA metabolic process (4.48e-3), translation (7.53e-5), heterocycle metabolic process (1.1e-2), ribosome (2.28e-2), nucleolus (2.28e-2)

Graded up compared to wild-type, no change in knockout

ORCP: mitochondrial envelope (1.71e-2)

URCP: translation (1.10e-2)

Graded down compared to wild-type

ORCP: Nucleolus (2.53e-7), Ribosome biogenesis (5.51e-6), RNA metabolic process (6.93e-3), plasma membrane (2.52e-2)

URCP: Cytoplasm (2.87e-4), protein binding (3.09e-2), response to stress (3.09e-2), mitochondrial envelope (4.48e-2), structural molecule activity (4.48e-2)

3. Non-catalytically associated genes (differentially expressed in knockout, no gradation)

ORCP: Conjugation (1.55e-4), plasma membrane (1.55e-4), extracellular region (3.08e-3), cell wall (6.16e-3), membrane (1.83e-2)

URCP: Cytoplasm (1.00e-5), DNA metabolic process (1.97e-2), protein modification process (1.97e-2), RNA binding (3.63e-2), nucleus (2.66e-2), triplet codon-amino acid adaptor activity (3.47e-2), response to stress (3.63e-2), ribosome (3.63e-2)

4. False negative genes

No functional enrichment/depletion of cellular processes detected.

5. Opposite genes

No functional enrichment/depletion of cellular processes detected.

| **Gene Category** | **ORCP** | **Yeast Gene IDs** |
| --- | --- | --- |
| Differentially expressed (DE) genes between wild-type & knockout | Cell wall | YDR055W YJL171C YIR039C YJR004C YNR044W YBR067C YLR042C YKL163W YLR194C YJL052W YLR040C YIL011W YOR382W YGR189C YMR008C YMR006C YDR077W |
|  | conjugation | YCL027W YBL016W YNR044W YJR004C YNL279W YBR083W YIL037C YHR005C YKL178C YFR008W YLR452C YJL157C YGL089C |
|  | Extra-cellular region | YDR055W YNR044W YBR067C YJR004C YKL163W YLR042C YPL123C YLR040C YIL011W YOR382W YGR189C YHR057C YMR006C YGL089C YDR077W |
|  | Plasma membrane | YPL265W YAR033W YDR055W YBR021W YGL053W YCL027W YNL279W YBL042C YLR194C YAR027W YMR319C YPR194C YML123C YLR214W YOL020W YOR101W YJL219W YMR008C YFL051C YLR452C YLR121C YAR031W YFL041W YBR068C YIR039C YPR124W YCL048W YIR032C YPL058C YHR005C YPR192W YOL156W YKL178C YGR121C YDR508C YLR413W YOL152W |
| DE genes between wild-type & knockout, up-regulated in knockout | Cell Wall | YIL011W YOR382W YJL171C YDR055W YIR039C YGR189C YKL163W YLR194C YMR008C YJL052W YDR077W |
| DE genes between wild-type & knockout, down-regulated in knockout | Nucleolus | YLR068W YIL127C YNL175C YGR280C YBL028C YNL124W YIL096C YDR021W YMR131C YBR247C YJL050W YJL033W YBR141C YJL109C YGL029W YGR159C YKL078W YLR145W YMR128W YAL059W YCR072C |
|  | Ribosome biogenesis | YBR267W YLR068W YGR280C YNL124W YNL112W YDR101C YDR021W YMR131C YBR247C YJL050W YHR197W YJL033W YJL109C YGL029W YLR059C YNL182C YGR159C YKL078W YLR145W YCR018C YMR128W YAL059W YCR072C |
|  | Conjugation | YKL178C YBL016W YCL027W YNR044W YJR004C YNL279W YBR083W YLR452C YIL037C YJL157C YGL089C YHR005C |
|  | Plasma membrane | YFL041W YBR021W YPR124W YCL027W YNL279W YBL042C YIR032C YHR005C YPR192W YMR319C YKL178C YML123C YLR214W YOL020W YOR101W YGR121C YLR452C YDR508C YLR413W YOL152W |
|  | Extracellular region | YHR057C YBR067C YNR044W YJR004C YLR042C YMR006C YGL089C YLR040C |
|  | RNA metabolic process | YNL141W YLR068W YJL050W YJL033W YJL109C YOL124C YNL182C YGR159C YLR145W YKL078W YMR128W YCR072C YBR267W YOL125W YOL066C YIL131C YGR280C YNL040W YNL124W YNL112W YDR021W YLR298C YMR131C YBR247C YHR197W YLR059C YGL029W YCR018C YAL059W YGR129W YDR465C |
| Graded genes compared to wild-type | Oxidoreductase activity | YPL171C YNL274C YER069W YKL107W YLL041C YML131W YGL055W YOR136W YJL052W YOR374W YNL037C YLR214W YIR036C YGR234W YIR038C YEL024W YJR096W YAL061W YLR460C YCL026C-B YIL155C YIL111W YMR118C YGR088W YBR026C YDL085W YCR102C YOR120W YOL152W |
| Graded up compared to wild-type | Oxidoreductase activity | YNL274C YPL171C YER069W YLL041C YML131W YKL107W YCL026C-B YIL155C YOR136W YJL052W YIL111W YOR374W YMR118C YGR088W YNL037C YBR026C YIR036C YDL085W YIR038C YEL024W YJR096W YAL061W YOR120W |
|  | Cellular protein catabolic process | YOR173W YFR053C YGR161C YIL155C YDR003W YPL002C YOR185C YGL156W YFR050C YLR178C YBR214W YKR098C YER054C YGR088W YJL020C YMR174C YDR358W YGL180W YIR038C YAL061W |
| Graded up compared to wild-type, no change in knockout | Mitochondrial envelope | YIL136W YDL142C YEL039C YJL161W YIL155C YJL066C YIL111W YER004W YMR118C YPL004C YDR178W YBR147W YDL085W YDR236C YIR038C YKL087C YEL024W |
| Graded down compared to wild-type | Nucleolus | YLR068W YIL127C YMR290C YNL175C YGR280C YBL028C YMR269W YDR021W YDL148C YMR131C YJL050W YJL109C YGL029W YGR159C YKL078W YLR145W YPL157W YMR128W YAL059W YGR245C |
|  | Ribosome biogenesis | YLR068W YMR290C YGR280C YNL112W YDR101C YMR269W YDR021W YLR074C YDL148C YMR131C YJL050W YHR197W YJL109C YGL029W YGR159C YKL078W YLR145W YPL157W YMR128W YAL059W YGR245C |
|  | RNA Metabolic process | YNL141W YLR068W YMR290C YDL201W YDL148C YJL050W YJL109C YGR159C YLR145W YKL078W YMR128W YOL066C YOL125W YIL131C YGR280C YNL112W YDR021W YMR269W YLR298C YMR131C YHR197W YGL029W YPL157W YAL059W YGR129W YDR465C |
|  | Plasma membrane | YBR294W YBR021W YOR273C YPR124W YGL255W YMR319C YHL016C YPR194C YML123C YLR214W YOL020W YGR121C YLR413W YOL152W |
| Non-catalytically associated genes (differentially expressed in knockout, no gradation) | Plasma membrane | YAR033W YFL041W YBR068C YGL053W YCL027W YNL279W YBL042C YCL048W YLR194C YPL058C YIR032C YHR005C YPR192W YOL156W YKL178C YOR101W YJL219W YFL051C YLR452C YLR121C YDR508C YAR031W |
|  | Conjugation | YKL178C YBL016W YCL027W YNR044W YJR004C YNL279W YBR083W YLR452C YIL037C YJL157C YGL089C YHR005C |
|  | Extracellular Region | YOR382W YGR189C YHR057C YBR067C YNR044W YJR004C YLR042C YGL089C YLR040C |
|  | Cell Wall | YOR382W YJL171C YGR189C YBR067C YNR044W YJR004C YLR042C YLR194C YLR040C |
|  | Membrane | YDR492W YGL053W YBL042C YLR040C YJL082W YJL037W YLR050C YAR035W YJL157C YBR222C YAR031W YNR065C YCL048W YIR032C YFL054C YCL021W-A YER100W YDR366C YGR189C YDL072C YDR508C YNR066C YAR033W YJL171C YPL156C YCL027W YJR004C YBR067C YNL279W YLR042C YLR194C YIL037C YPL057C YEL004W YOR382W YLR145W YOR101W YFL051C YJL219W YLR121C YLR452C YHL026C YFL041W YBR068C YER060W YNR044W YDR034C-A YPL058C YHR005C YPR192W YDR275W YOL156W YKL178C YDR218C |

**References**

1. Maere S, Heymans K, Kuiper M (2005) BiNGO: a Cytoscape plugin to assess overrepresentation of gene ontology categories in biological networks. Bioinformatics 21: 3448-3449.
